# Supplementary material for: Significance of NatB-mediated N-terminal acetylation of auxin biosynthetic enzymes in maintaining auxin homeostasis in Arabidopsis thaliana
Source: Commun Biol. 2022 Dec 22;5:1410. doi: 10.1038/s42003-022-04313-9 (PMC9780221; doi:10.1038/s42003-022-04313-9)
Supplement: Supplementary file 2 — Supplementary Information [file 42003_2022_4313_MOESM2_ESM.pdf]

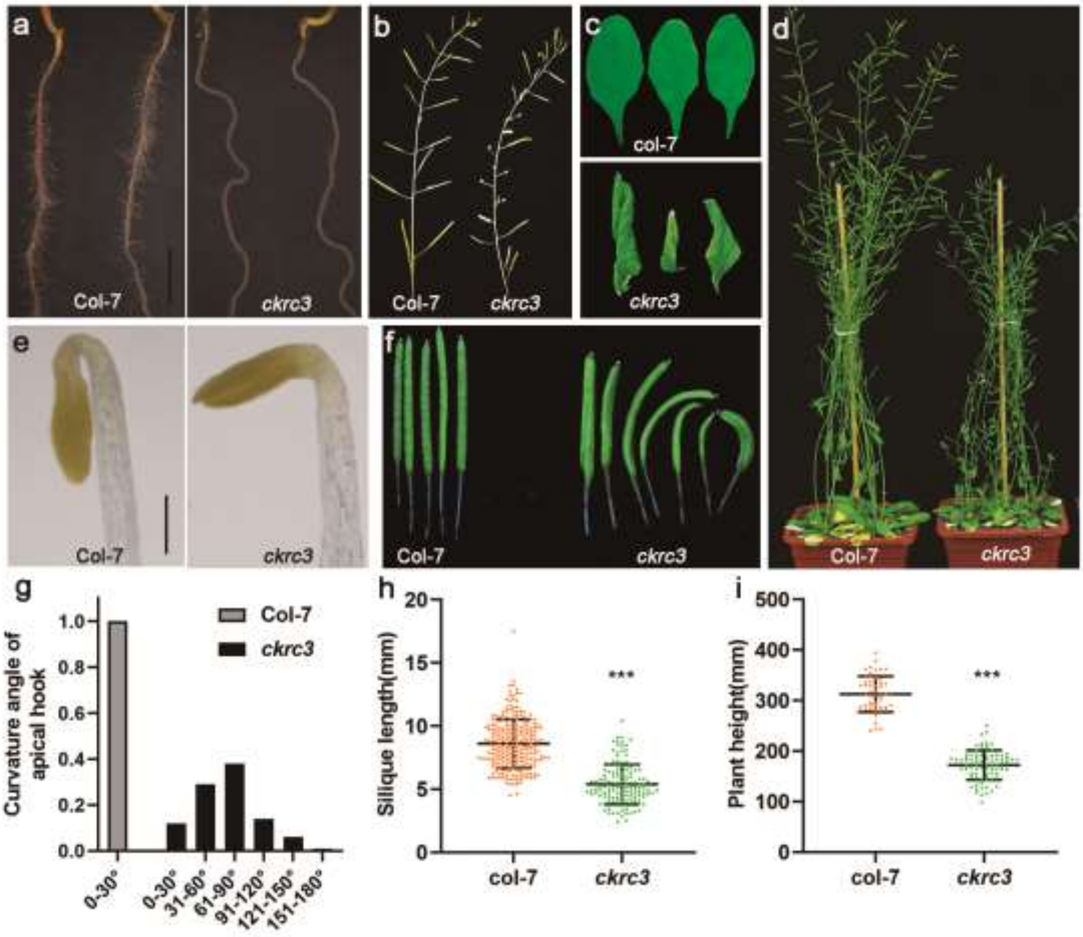

Supplementary Figure 1. The pleiotropic morphology of *ckrc3* mutant. a, Comparison of the root hairs between Col-7 and *ckrc3*, the seedlings were grown on MS medium for 7 days, the bar=2 mm. **b**, The abortive siliques of *ckrc3*. **c**, Leaf reticulation. **d, f**, Plant height and siliques. **e**, The apical hook of 3 days etiolated seedlings. **g,** quantification of hook curvature of 3 days etiolated seedlings grown on MS medium. **h-i**, Quantification of siliques length (n ≥ 142) and plant height (n ≥ 52). Data are presented as mean ± S.D., $^{***}P$<0.001 according to ANOVA followed by Tukey's multiple comparison tests.

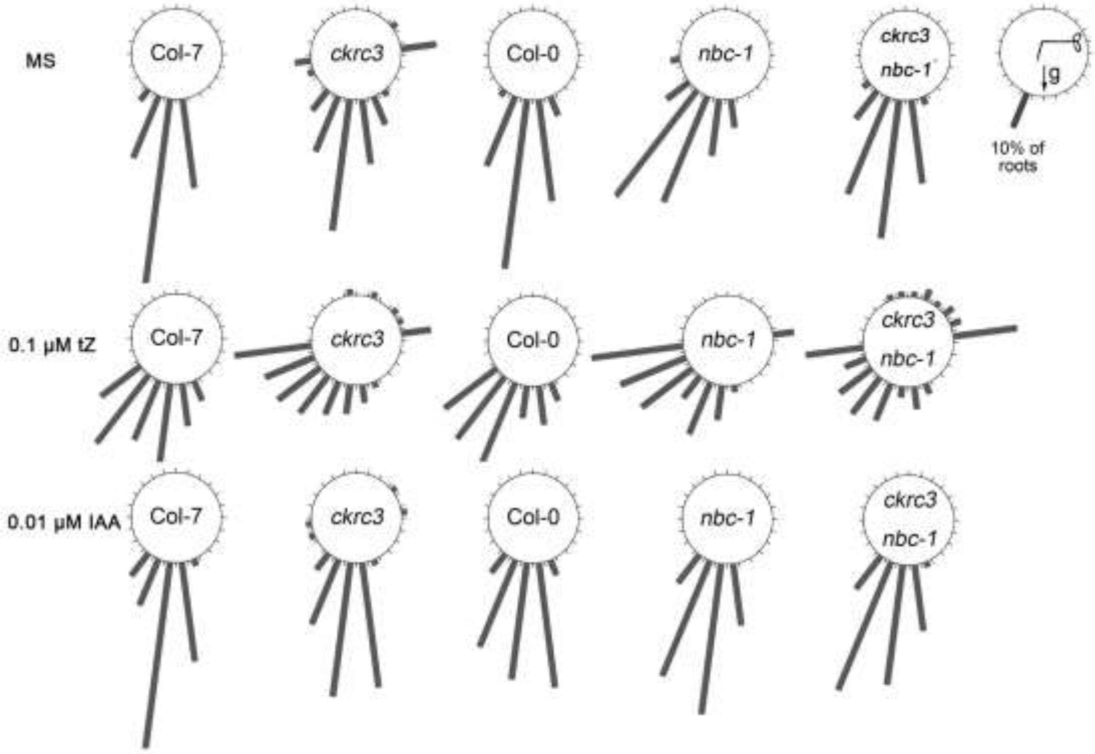

Supplementary Figure 2. Effects of exogenous IAA and tZ on gravitropic response; n ≥ 39 per genotype/treatment, *ckrc3*, *nbc-1*, *ckrc3 nbc-1* mutants exhibited defective gravitropic response on MS, more serious on MS containing 0.1μM tZ and were recovered on MS with 0.01μM IAA.

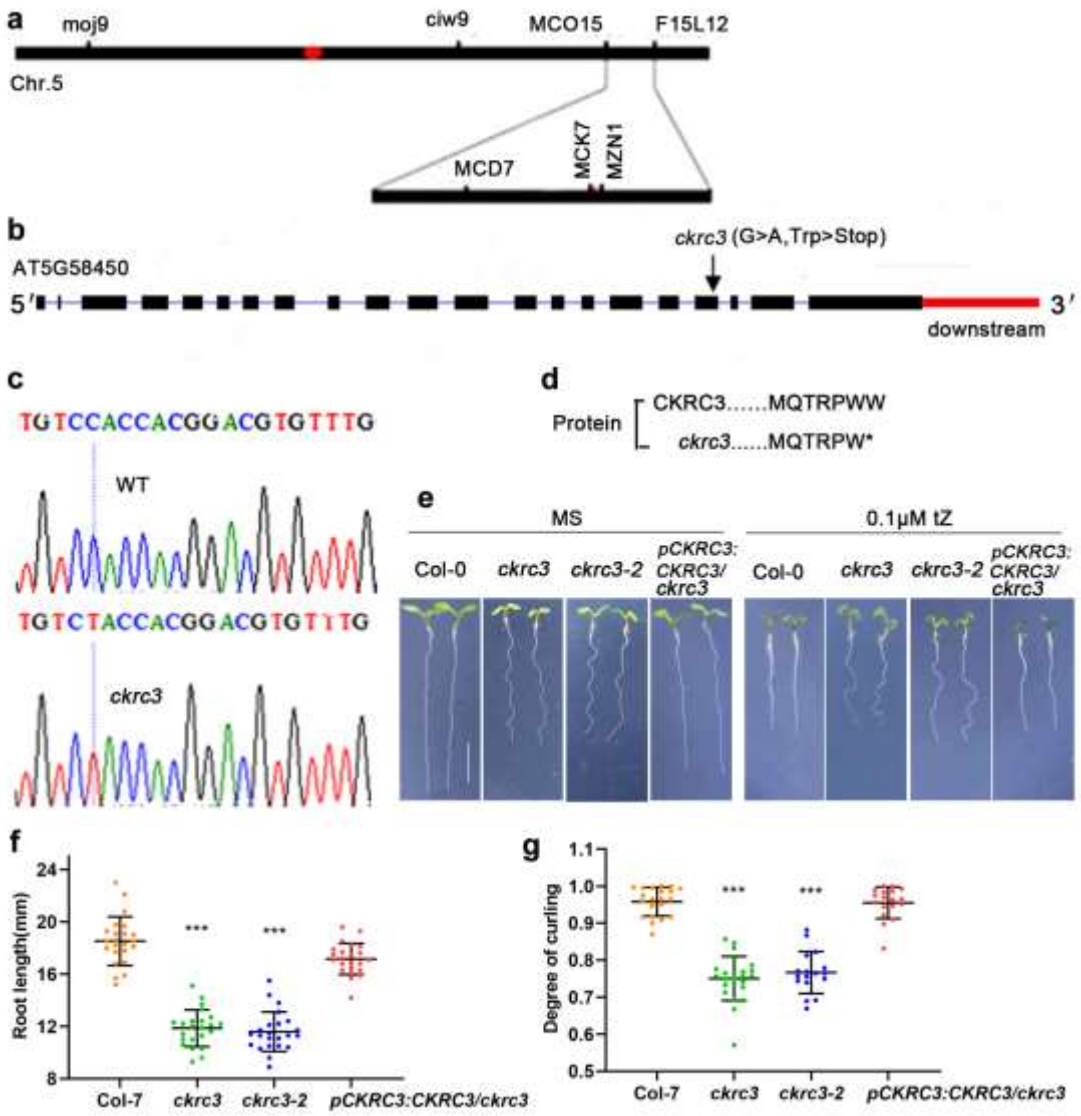

Supplementary Figure 3. Structure of the *CKRC*3 gene, its allele mutant and molecular complementation of the *ckrc3* mutant. **a-d**, Map-based cloning of *CKRC3*. **e-g**, Genetic allelic analysis and molecular complementation of *ckrc3* mutant. Data are presented as mean ± S.D., $^{***}P<0.001$ according to ANOVA followed by Tukey's multiple comparison tests. (f, n = 23; g, n ≥ 17).

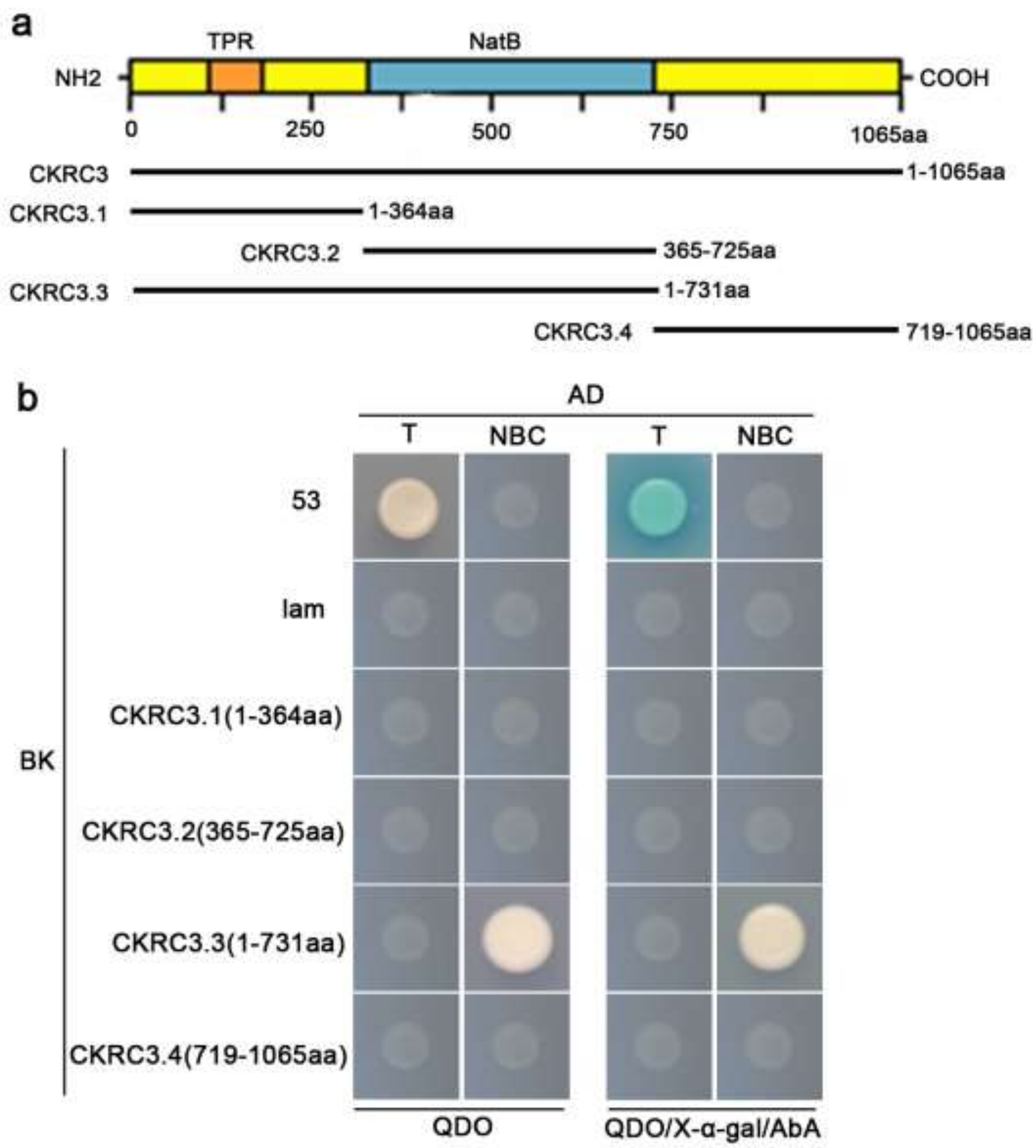

Supplementary Figure 4. Interaction between CKRC3 and NBC. a, The CKRC3 protein containing several tetratricopeptide repetitions (TPR; residues 89 to 181aa) and a NatB domain (residues 365-725), with the four truncated versions drawn below. **b**, Y2H results.

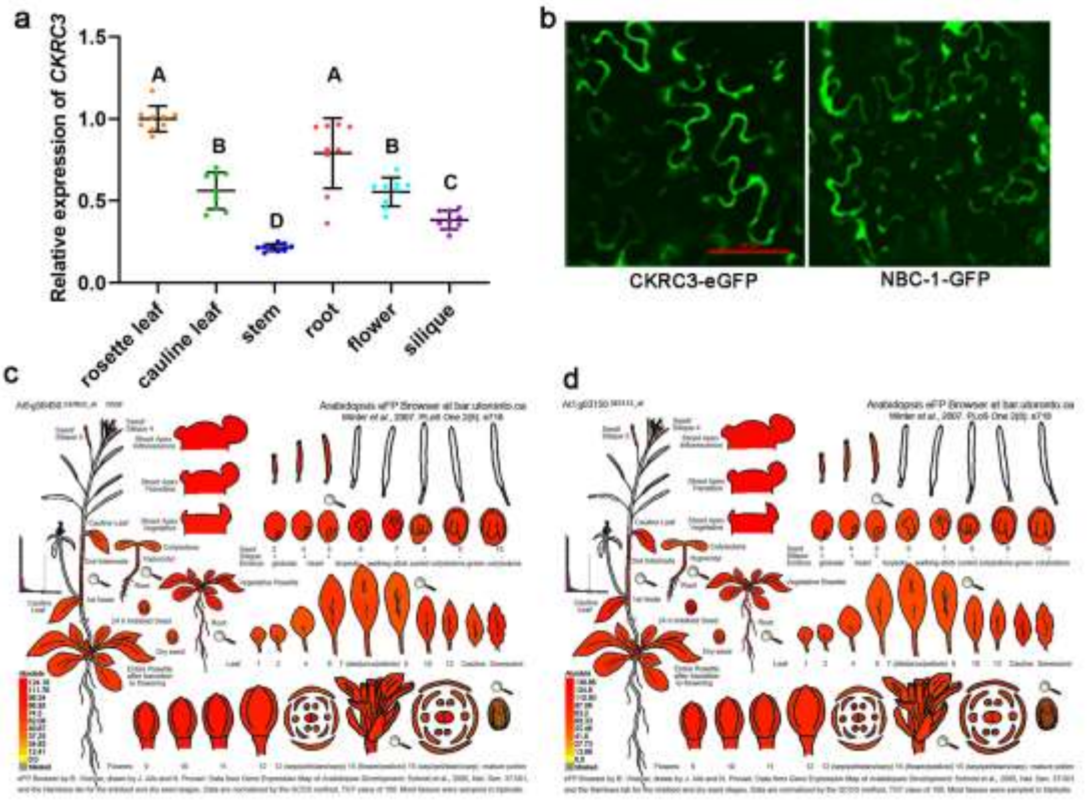

Supplementary Figure 5. Tissues expression pattern and subcellular localization. **a**, *CKRC3* transcript levels in different tissues, as determined by qRT-PCR; Data are presented as mean ± S.D. of three independent experiments, different capital letters indicate significant differences at $P<0.01$ according to ANOVA followed by Tukey's multiple comparison tests. **b**, Subcellular localization of CKRC3 and NBC, bar=100 μM. **c-d**, Data from the open access eFP Browser (http://bar.utoronto.ca/efp2/) showing the wide expressions of *CKRC3/TCU2* (**c**) and *NBC* (**d**) throughout plant development.

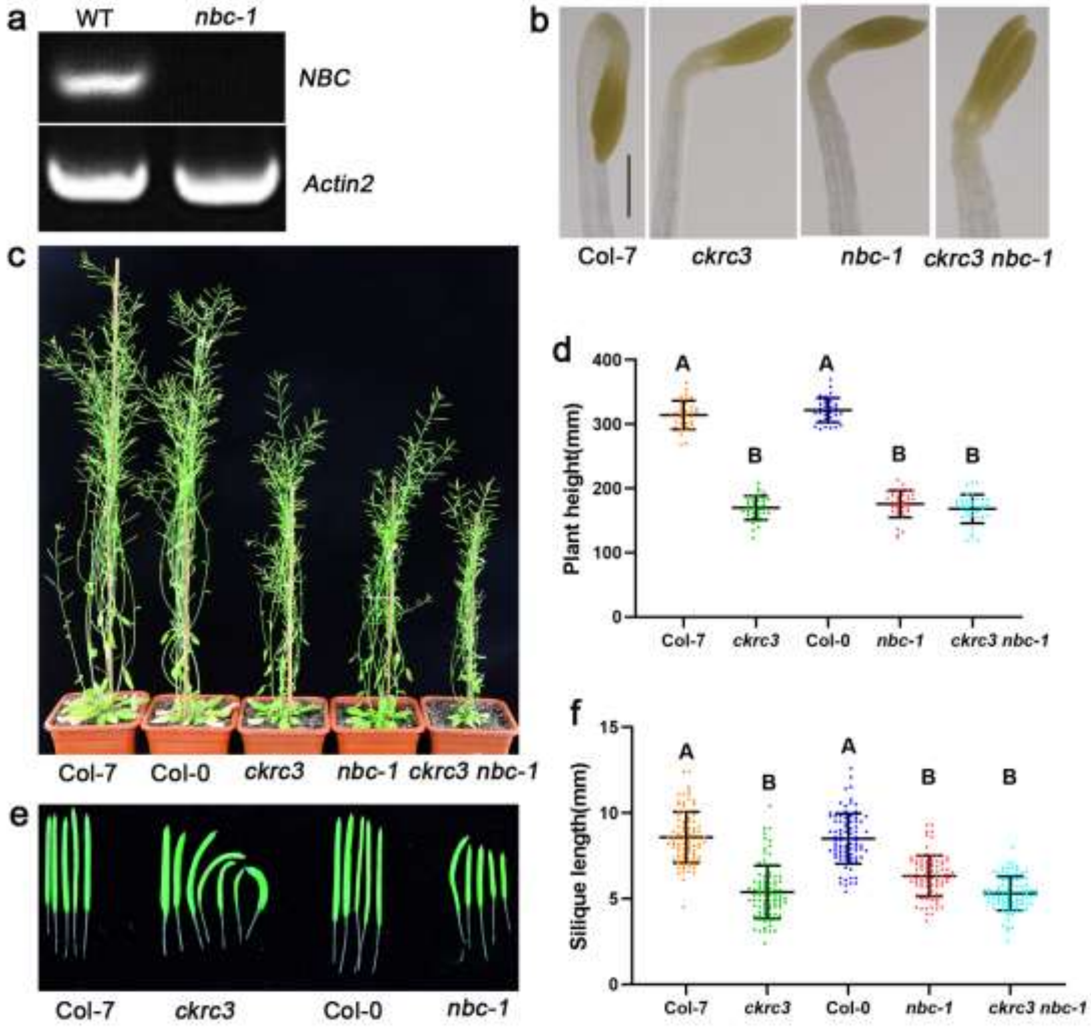

Supplementary Figure 6. The transcript levels of *NBC* and the phenotypes of *nbc-1*, *ckrc3 nbc-1* mutants. **a**, Semi-quantitative RT-PCR. **b**, The apical hook of 3 days etiolated seedling grown on MS medium, bar=2 mm. **c, e**, The phenotypes of plant height and siliques. **d, f**, Quantification of plant height (n = 36) and siliques length (n ≥ 89). Data are presented as mean ± S.D., different capital letters indicate significant differences at *P*<0.01 according to ANOVA followed by Tukey's multiple comparison tests.

a

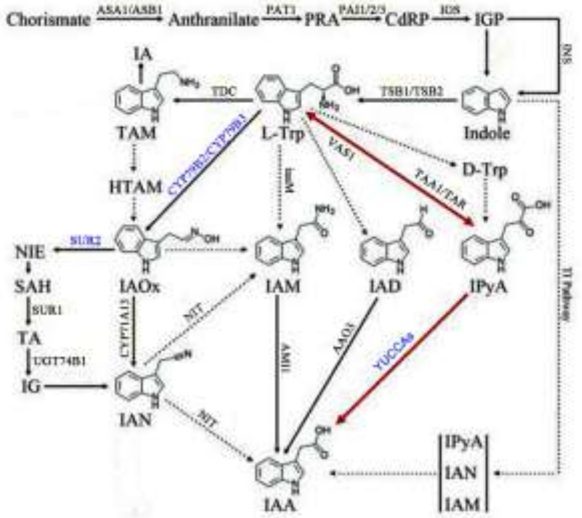

b

YUCCA1	NH2-MESHP • • • • • FTSKS-COOH	414Aa	TRP3	NH2-MAIAF • • • • • KSALL-COOH	312Aa
YUCCA2	NH2-MEFVT • • • • • RPQHC-COOH	415Aa	CYP79B2	NH2-MNTFT • • • • • YPTVK-COOH	541Aa
YUCCA3	NH2-MYGNN • • • • • CISHF-COOH	437Aa	CYP79B3	NH2-MDTLA • • • • • YPMVK-COOH	543Aa
YUCCA4	NH2-MGTCR • • • • • HFNKS-COOH	411Aa	AMI1	NH2-MATNN • • • • • LAAFI-COOH	425Aa
YUCCA5	NH2-MENMF • • • • • CISVA-COOH	424Aa	SUR1	NH2-MSEEQ • • • • • NNLEM-COOH	462Aa
YUCCA6	NH2-MEGKL • • • • • DCGGN-COOH	426Aa	SUR2	NH2-MDLLL • • • • • PTKHI-COOH	499Aa
YUCCA7	NH2-MCNNN • • • • • CISHF-COOH	431Aa	UGT74B1	NH2-MAETT • • • • • ESLGK-COOH	460Aa
YUCCA8	NH2-MENMF • • • • • ISQQF-COOH	426Aa	AAO1	NH2-MGEKA • • • • • QRKNV-COOH	1368Aa
YUCCA9	NH2-MENMF • • • • • CISVA-COOH	421Aa	AAO2	NH2-MSLVF • • • • • DNSNL-COOH	1321Aa
YUCCA10	NH2-METVV • • • • • TLKNN-COOH	383Aa	AAO3	NH2-MDLEF • • • • • KIKGQ-COOH	1332Aa
YUCCA11	NH2-MEKEI • • • • • NKLSK-COOH	391Aa	AAO4	NH2-MAGDD • • • • • WKTYP-COOH	1337Aa
CKRC1	NH2-MVKLE • • • • • KSIDL-COOH	391Aa	NIT1	NH2-MSSTK • • • • • DDSNK-COOH	346Aa
VAS1	NH2-MGSFG • • • • • HGMVE-COOH	394Aa	NIT 2	NH2-MSTSE • • • • • DDSNK-COOH	339Aa
WEI2	NH2-MSSSM • • • • • AFVKK-COOH	624Aa	NIT 3	NH2-MSSTE • • • • • DESNK-COOH	346Aa
WEI7	NH2-MAAST • • • • • SEKLT-COOH	289Aa	CYP71B15	NH2-MSVFL • • • • • QVLHH-COOH	490Aa
TRP1	NH2-MVIAV • • • • • AQKSQ-COOH	444Aa	CYP71A13	NH2-MSNIQ • • • • • PSSVV-COOH	503Aa
TRP2	NH2-MAASG • • • • • KYLDV-COOH	470Aa	MYR	NH2-MAIPK • • • • • KFLAK-COOH	511Aa

Supplementary Figure 7. The proposed IAA biosynthesis pathways and N-terminal peptides of auxin biosynthetic enzymes in Arabidopsis. a, The IAA biosynthesis pathways[1]. **b,** amino sequence of NatB substrate signatures are indicated by arrows.

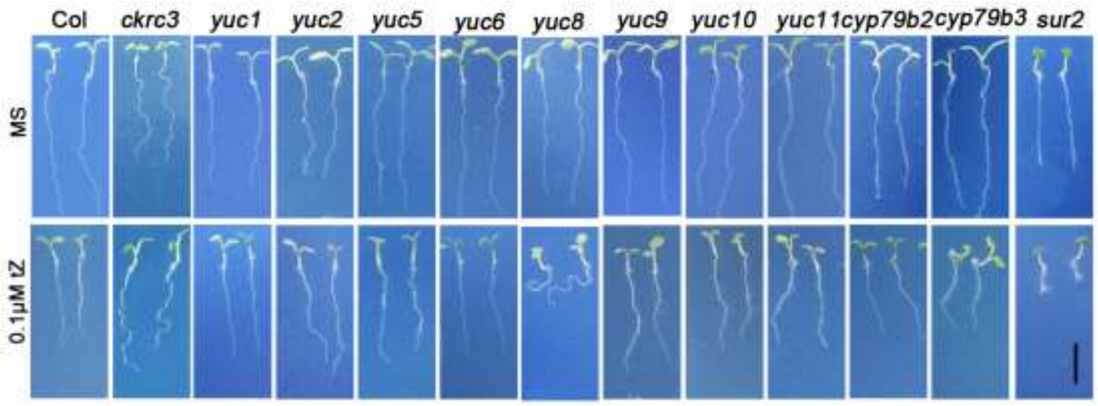

Supplementary Figure 8. Root phenotypes of WT and *yuc* mutants on MS and 0.1µM tZ medium. Bar=5 mm

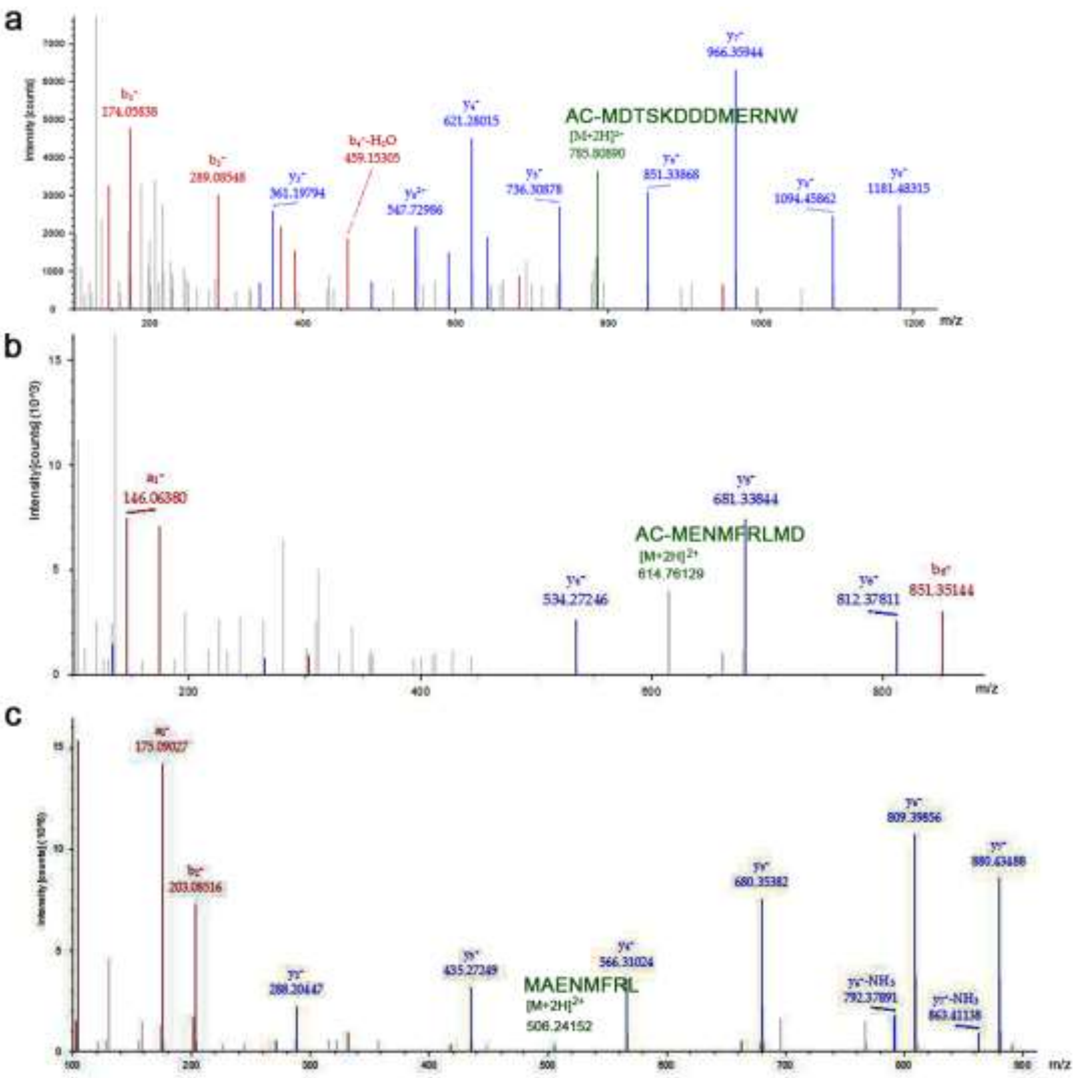

Supplementary Figure 9. NatB had enzyme activity on YUC8 in *vitro*. Annotated LC-MS/MS spectrum of the peptides corresponding to SNC1 and YUC8. NTA modification were detected in positive control N-terminal peptide of SNC1 protein (**a**) and that of YUC8(N-ME) (**b**), but not in the mutated YUC8(N-MAE) **(c)**.

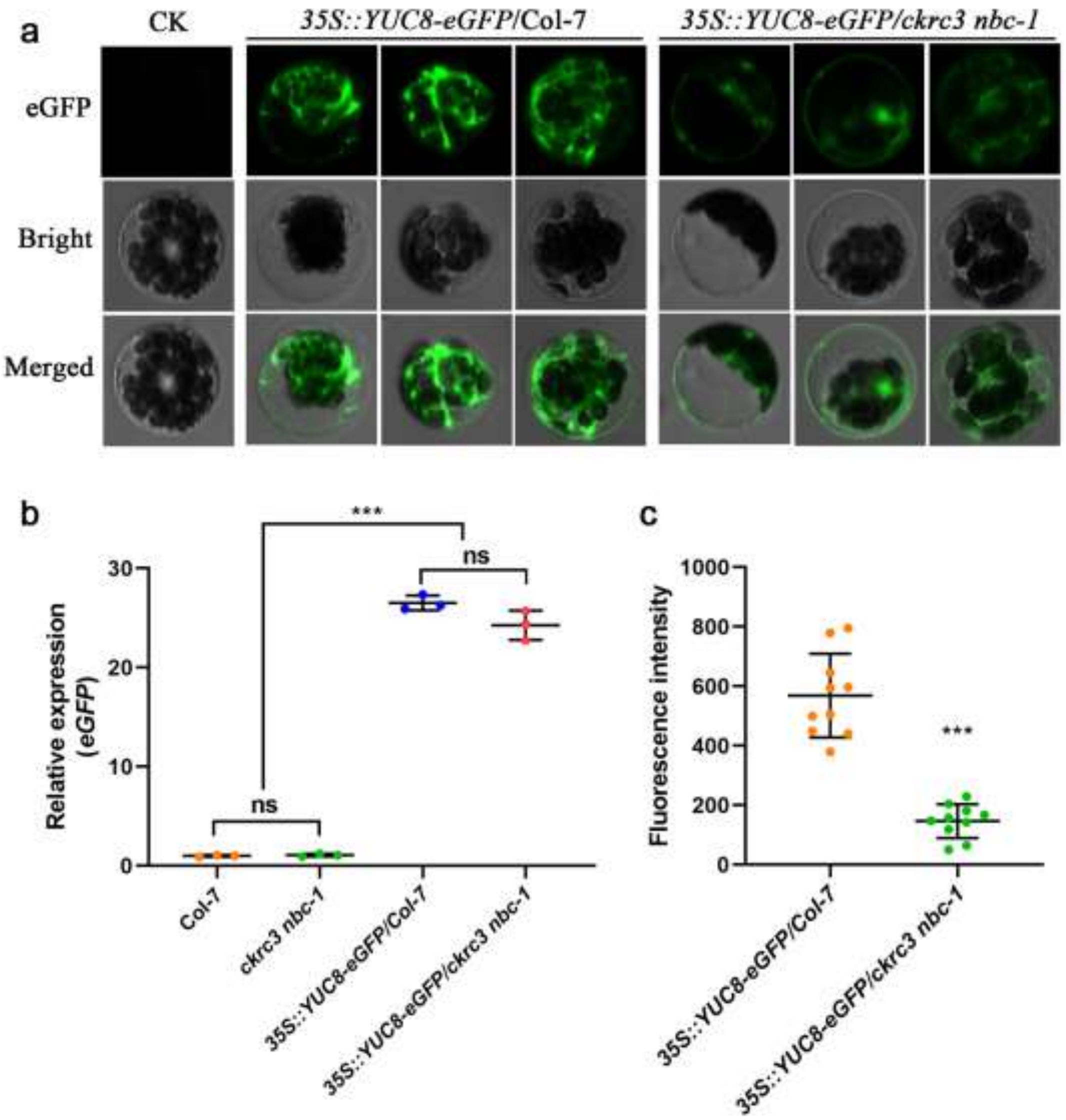

Supplementary Figure 10. YUC8-eGFP fusion protein levels were determined after transient expression in protoplasts. a, eGFP fluorescence of protoplasts. **b**, *eGFP* transcript levels. Data are presented as mean ± S.D. of three experimental repeats, $^{***}P<0.001$ according to ANOVA followed by Tukey's multiple comparison tests. **c**, Quantification results of fluorescence intensity. Data are presented as mean ± SD., n=10, $^{***}P<0.001$ according to ANOVA followed by Tukey's multiple comparison tests.

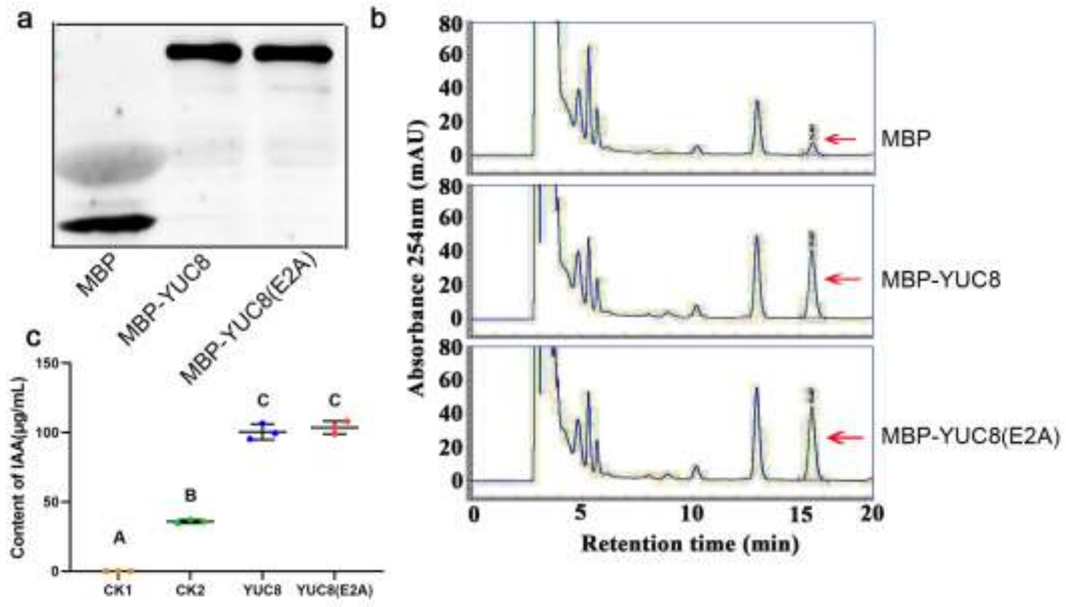

Supplementary Figure 11. Substitution of the second residue does not significantly affect the enzyme activity of YUC8. a, Western blot of MBP, MBP-YUC8 and MBP-YUC8(E2A) fusion proteins. **b**, HPLC chromatograms of the products of the in vitro YUC reactions catalyzed by the purified MBP-YUC8 protein or its mutant forms MBP-YUC8(E2A) and control (MBP). **c**, Quantification of enzyme activities of YUC8 and its mutant forms YUC8(E2A), without IPyA (substrate) as CK1, and empty vector MBP as CK2. Data are presented as mean ± S.D. of three experimental repeats, different capital letters indicate significant differences at *P*<0.01 according to ANOVA followed by Tukey's multiple comparison tests.

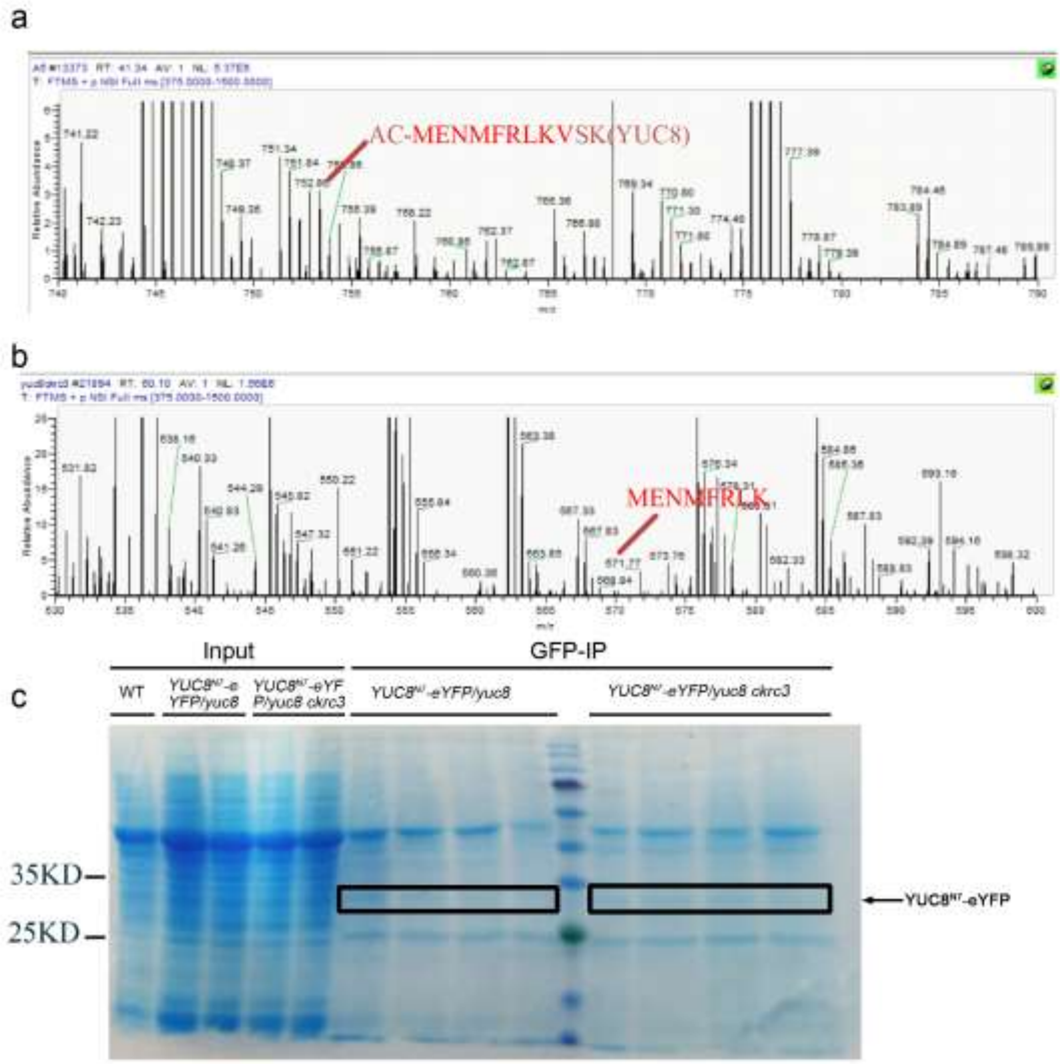

Supplementary Figure 12. Initiator Met (iMet) of YUC8 undergoes NTA *in planta*. a, MS spectrum of YUC8^{N7}-eYFP, showing acetylation on its iMet in *yuc8* single mutant (**a**) but not detected in *yuc8 ckrc3* double mutant (**b**). **c**, Isolated proteins were enriched by IP and subjected to LC-MS/MS analysis.

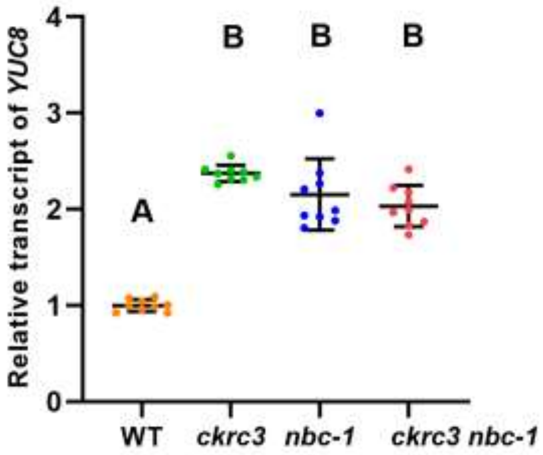

Supplementary Figure 13. ***YUC8* expression levels determined by qRT-PCR**, Data are presented as mean ± S.D. of three independent experiments, different capital letters indicate significant differences at *P*<0.01 according to ANOVA followed by Tukey's multiple comparison tests.

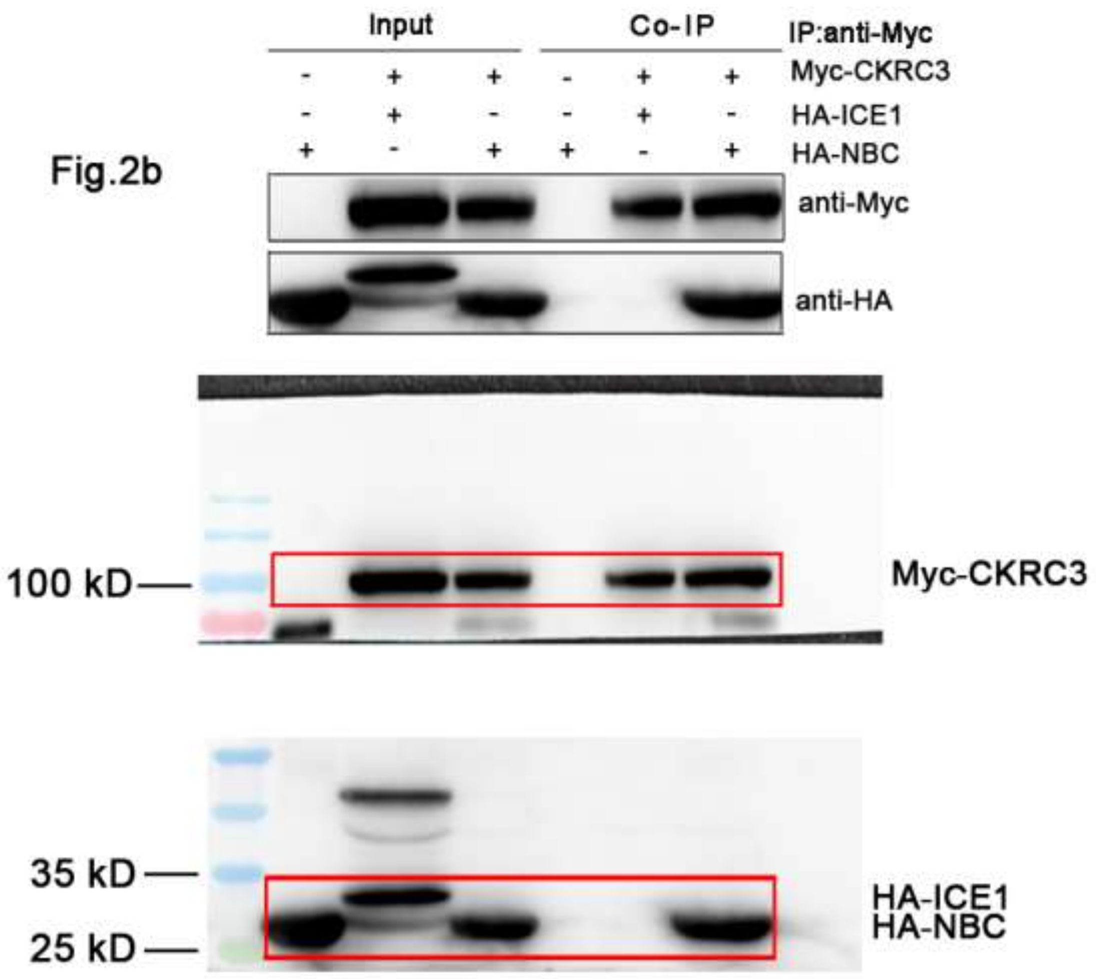

Supplementary Figure 14. Uncropped Western-blots images corresponding to Figure 2b.

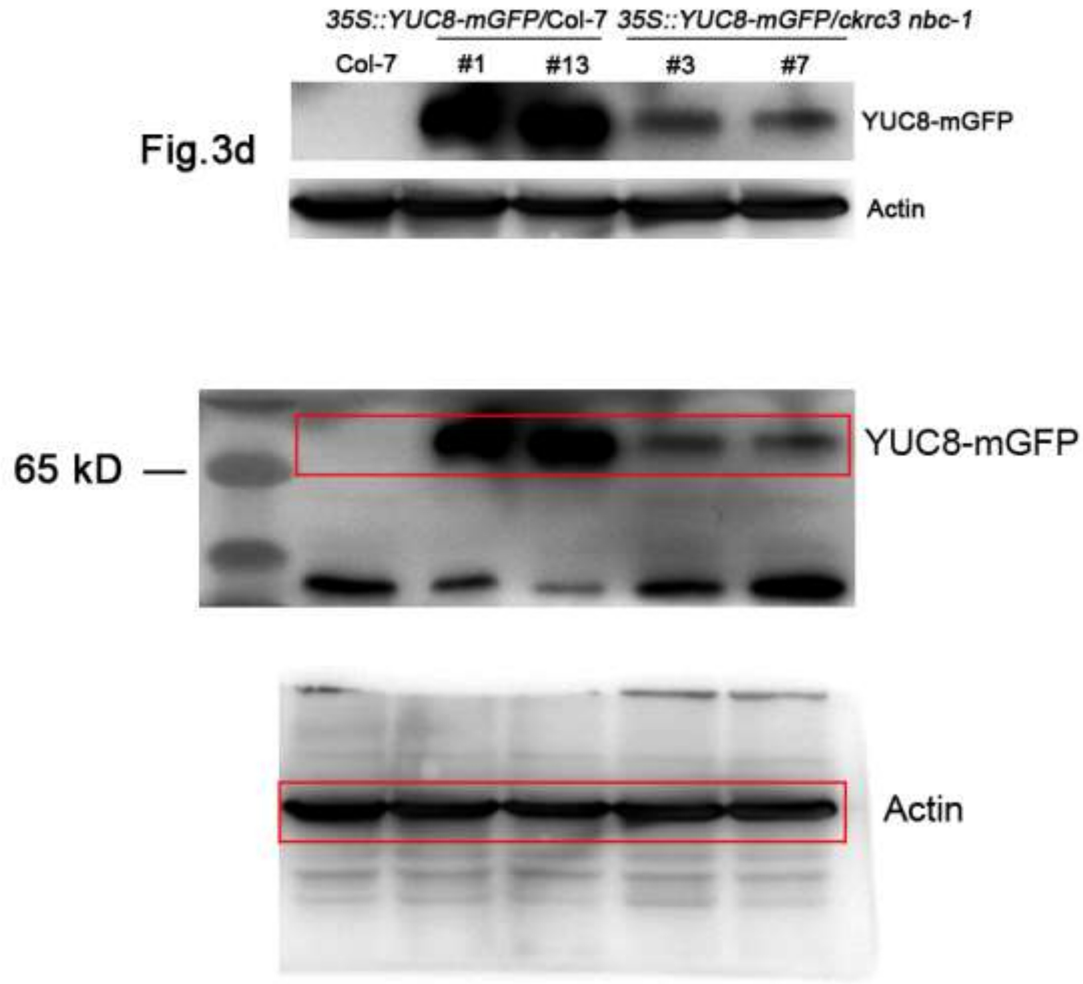

Supplementary Figure 15. Uncropped Western-blots images corresponding to Figure 3d.

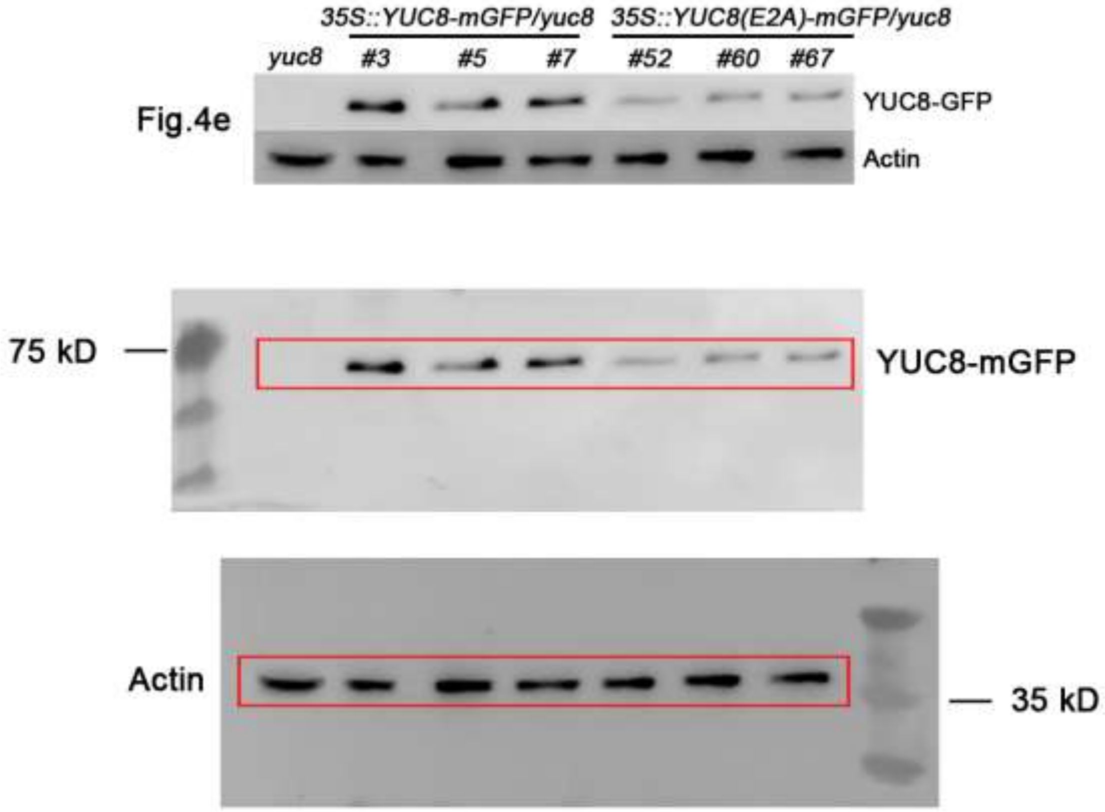

Supplementary Figure 16. Uncropped Western-blots images corresponding to Figure 4e.

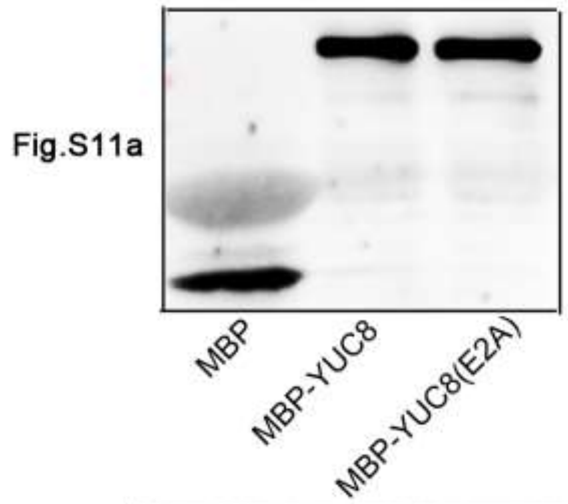

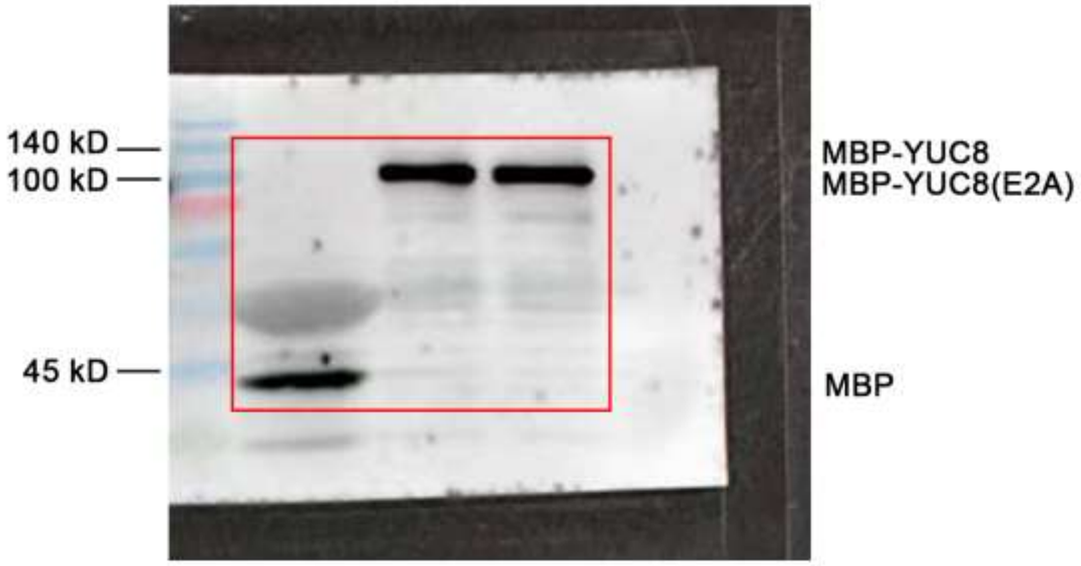

Supplementary Figure 17. Uncropped Western-blots images corresponding to Figure S11a.

Supplementary Table 1. LC-MS/MS analysis of the N-terminal peptides of different proteins

Protein	N-terminal isoforms detected by MS	Number of MS hit	Charge	MH+ [Da]
SNC1 [Positive control 1]	AC-MDTSKDDDMERWG	4	2	1627.64
	AC-MDTSKDDDMERW	2	2	1570.61
	AC-MDTSKDDDMERWGRP	2	3	1880.79
YUC8	AC-MENMFRLMD	1	2	1228.515
	AC-ENMFRLMDQR	1	2	1381.635
	AC-ENMFRLM	1	2	982.451
	AC-ENMFRL	2	2	851.408
YUC8(N-MAE) [Negative control 1)	MAENMFRL	5	2	1011.476
	MAENMFRLMDQ	1	2	1385.602
	MAENMFRLM	9	2	1142.516
SNC1 [Positive control 2]	AC-MDTSKDDDMER	3	3	1384.541
	AC-MDTSKDDDMER	7	2	1384.541
	AC-MDTSKDDDMER (M9-Oxidation)	1	3	1400.533
	AC-MDTSKDDDMER (M9-Oxidation)	1	2	1400.533
YUC1	AC-MESHPHNKTDR	4	4	1393.628
	AC-MESHPHNKTDR	6	3	1393.628
	AC-MESHPHNKTDR	1	2	1393.628
	AC-MESHPHNKTDR (S3-Phospho)	1	3	1473.588
	AC-MESHPHNK	1	2	1021.456
YUC2	AC-MEFVTETLGKR	7	3	1352.692
	AC-MEFVTETLGKR	10	2	1352.692
	AC-MEFVTETLGKR (T5-Phospho)	1	2	1432.652
	AC-MEFVTETLGKRWGRPVGR (T5-Phospho)	1	4	2241.115
	AC-MEFVTETLGK	4	2	1196.591
YUC3 [Negative control 2)	MYGNNNKK	1	2	968.464
YUC5	AC-MENMFR	34	2	869.366
	AC-MENMFR (M4-Oxidation)	4	2	885.362
YUC6	AC-MDFCWKR (C4-Carbamidomethyl)	3	3	1084.475
	AC-MDFCWKR (C4-Carbamidomethyl)	10	2	1084.475
	AC-MDFCWK (C4-Carbamidomethyl)	11	2	928.372
YUC9	AC-MENMFR	29	2	869.366
	AC-MENMFR (M4-Oxidation)	2	2	885.362

Supplementary Table 2. Mutants used

Mutant	Stock No.	Ecotype background	Mutation type	Source	Mutation gene
ckrc3		Col-7	point mutation	This lab	*AT5G58450*
ckrc3-2	N862072	Col-0	T-DNA	uNASC	*AT5G58450*
nbc-1	SAIL_323_B05	Col-0	T-DNA	uNASC	*AT1G03150*
sur2	SALK_012581	Col-0	T-DNA	ABRC	*AT4G31500*
yuc1	N655809	Col-0	T-DNA	uNASC	*AT4G32540*
yuc2	N659779	Col-0	T-DNA	uNASC	*AT4G13260*
yuc5	CSHL_GT6160	Ler	T-DNA	Zhao	*AT5G43890*
yuc6	N663363	Col-0	T-DNA	uNASC	*AT5G43890*
yuc8		Col-0	deletion	This lab	*AT4G28720*
yuc9	SAIL_762-D07	Col-0	T-DNA	Zhao	*AT1G04180*
yuc10	FLAG_599G05	Col-0	T-DNA	Chen	*AT1G48910*
yuc11	N573485	Col-0	T-DNA	uNASC	*AT1G21430*
ckrc1		Col-0	T-DNA	This lab	*AT1G70560*
cyp79b2	SALK_130570	Col-0	T-DNA	ABRC	*AT4G39950*
cyp79b3	N851035	Col-0	T-DNA	uNASC	*AT2G22330*

Supplementary Table 3. PCR primers

**Name**	**Sequence**
*ckrc3*F	ACATGGCTTTGATAGGTCTC
*ckrc3*R	TCATTTCTACAGCGTCACTT
*nbc-1*LP	CCATGCTTCCTATGCATCTTC
*nbc-1*RP	GTCGGTTAGGGCTTTTGAATC
PLB1	GCCTTTTCAGAAATGGATAAATAGCCTTGCTTCC
*sur2*LP	TGCGAAGTCAATAGGGACATC
*sur2*RP	GCTCACTTAGATCAACGGTGC
LBb1.3	ATTTTGCCGATTTCGGAAC
*yuc8*LP	ATTCTGCATTTGGTTCCACAC
*yuc8*RP	GACTCACTCTTCGACACGGTC
LBb1.3	ATTTTGCCGATTTCGGAAC
qRT-YUC8F	AAATACGGTTTGAAACGACCAG
qRT-YUC8R	TCGACTTTGTTTCCGTTAAACC
qRT-CKRC3F	CTGGCTGAGGGTTTACTTAAGA
qRT-CKRC3R	CATCAACAGCAGCAGAATAGTC
qRT-NBCF	GTCACTGTCTCTCCAGAATACC
qRT-NBCR	GCGTAAAACCCGCCTATAAATT
qRT-ACT8F	TGTGACAATGGTACTGGAATGG
qRT-ACT8R	TTGGATTGTGCTTCATCACC
nbc1(semi-q)-F	CCACCTCACTGAAACATTCAAT
nbc1(semi-q)-R	CCTTCCTCATATCTAATCCATC
pGBK-CKRC3-F	CATGGAGGCCGAATTCATGCGCAGGTGGGGACTATGG
pGBK-CKRC3-R	GCCGCTGCAGGTCGACTCAAACATGGGACATCTGTTGCTTG
pGAD-NBC-F	CAGGCCAGTGAATTCATGACGACGATACGTAGATTCAGC
pGAD-NBC-R	CGATGCCCACCCGGGTTAATCATACTCTAATTCATCAGG
Myc-CKRC3F	GACTCTTGACCATGGTCATCATGGAGGAGCAGAAGCTG
Myc-CKRC3R	TTCGAGCTGGTCACCGTCGACTCAAACATGGGACATC
HA-NBCF	GACTCTTGACCATGGTCATCATGGAGTACCCATACGACG
HA-NBCR	TTCGAGCTGGTCACCTTAATCATACTCTAATTCATCAGG
E2-NBC-F	TTTCAGGGCGCCATGGTCATGACGACGATACGTAGATTCAGCTG
E2-NBC-R	CGAGGAATTCGGATCCTTAATCATACTCTAATTCATCAGGTGTG
E2-YUC8-F	TTCAGGGCGCCATGGTCATGGAGAATATGTTTCGTTTGAT
E2-YUC8-R	GTGCTCGAGGAATTCGAACTGTTGAGAGATACACCTTCG
E2-YUC8(E2A)-F	TTCAGGGCGCCATGGTCATGGCGAATATGTTTCGTTTGAT
E2-YUC8(E2A)-R	GTGCTCGAGGAATTCGAACTGTTGAGAGATACACCTTCG
YUC8-mgfp-F	CGGGGGACTCTTGACCATGGAGAATATGTTTCGTTTGATGG
YUC8-mgfp-R	GTCAGATCTACCATGCTGAACTGTTGAGAGATACACCTTCG
YUC8(E2A)-mgfp-F	CGGGGGACTCTTGACATGGCGAATATGTTTCGTTTGATGG
YUC8(E2A)-mgfp-R	GTCAGATCTACCATGCTGAACTGTTGAGAGATACACCTTCG
YUC8-eGFP-F	TTTGGAGAGGACAGGGTACCATGGAGAATATGTTTCGTTTGATGG
YUC8-eGFP-R	CCATGTCGACTCTAGAGAACTGTTGAGAGATACACCTTCG
YUC8(E2A)-eGFP-F	TTTGGAGAGGACAGGGTACCATGGCGAATATGTTTCGTTTGATGG
YUC8(E2A)-eGFP-R	CCATGTCGACTCTAGAGAACTGTTGAGAGATACACCTTCG

References:

1 Di, D.-W., Zhang, C., Luo, P., An, C.-W. & Guo, G.-Q. The biosynthesis of auxin: how many paths truly lead to IAA? *Plant Growth Regulation* **78**, 275-285, doi:10.1007/s10725-015-0103-5 (2016).
