# Supplementary material for: Significance of NatB-mediated N-terminal acetylation of auxin biosynthetic enzymes in maintaining auxin homeostasis in Arabidopsis thaliana
Source: Commun Biol. 2022 Dec 22;5:1410. doi: 10.1038/s42003-022-04313-9 (PMC9780221; doi:10.1038/s42003-022-04313-9)
Supplement: Supplementary file 4 — Supplementary Data [file 42003_2022_4313_MOESM4_ESM.pdf]

data.Fig.1b

	MS	0.1 µM tZ		MS	0.1 µM tZ
col-7	0.924	1.000	ckrc3	0.834	0.779
	0.984	0.909		0.692	0.808
	0.970	0.997		0.854	0.703
	0.992	1.000		0.831	0.777
	1.000	0.987		0.799	0.647
	1.000	1.000		0.776	0.647
	0.956	1.000		0.736	0.701
	1.000	1.000		0.625	0.620
	0.898	0.950		0.696	0.683
	1.000	1.000		0.806	0.683
	0.985	0.977		0.576	0.763
	1.000	0.988		0.675	0.684
	0.954	0.969		0.770	0.758
	0.957	1.000		0.697	0.545
	0.919	0.992		0.752	0.839
	1.000	1.000		0.823	0.710
	1.000	1.000		0.735	0.740
	0.974	1.000		0.670	0.754
	0.962	0.914		0.774	0.645
	0.983	1.000		0.738	0.701
	1.000	1.000		0.806	0.731
		0.981		0.779	0.703
		1.000		0.838	0.745
		0.987		0.772	0.709
		1.000		0.805	0.709
		0.951			0.718
		1.000			0.603
		0.970			0.729
		1.000			0.689
		0.976			0.716
		1.000			0.650
		0.985			0.716
		1.000			0.701

data.Fig.1c

Col-7	ckrc3	sur2	ckrc3 sur2
21.6	13.7	17.1	21.5
21.9	14.1	14.9	20.6
19.9	12.4	15.7	22.3
20.7	15.2	14.0	20.8
20.4	13.5	11.7	18.8
20.5	14.4	12.5	18.8
22.2	13.3	10.3	18.7
20.4	14.9	13.4	19.8
21.1	13.3	17.5	21.9
19.6	14.6	16.0	19.3
22.7	15.4	16.3	21.4
21.1	13.6	14.2	20.0
22.7	14.5	16.0	19.7
20.2	13.8	15.8	20.3
22.0	12.9	13.4	20.8
22.9	14.9	14.6	21.0
21.2	12.9	14.1	20.0
22.0	14.2	15.4	19.9
20.5	14.1	17.7	22.7
21.0	14.1	15.8	17.7
21.7	13.1	14.6	20.7
21.9			20.5
			22.9
			19.8
			20.7
			21.6
			21.0
			20.0
			19.7
			23.1
			23.3
			21.5
			22.5
			21.4
			20.7

data.Fig.1d

Col-7	ckrc3	sur2	ckrc3 sur2
0.991	0.834	1.000	0.889
0.927	0.693	0.990	0.901
0.917	0.866	0.988	0.953
1.000	0.834	0.961	0.918
0.997	0.786	0.948	1.000
1.000	0.769	0.917	0.953
0.950	0.728	1.000	0.917
1.000	0.616	1.000	0.989
0.890	0.693	0.962	1.000
0.958	0.801	1.001	0.990
0.962	0.572	1.000	0.982
1.000	0.668	1.000	0.948
0.948	0.769	1.000	0.997
0.952	0.693	0.991	1.000
0.910	0.750	1.000	0.973
1.001	0.819	0.984	0.962
1.000	0.734	1.000	1.000
0.901	0.667	0.979	0.995
0.961	0.769	0.999	1.000
1.000	0.727	0.989	0.931
1.000	0.801		1.000
	0.770		0.978
	0.834		0.956
	0.769		0.917
	0.800		1.000
			0.985
			1.000

data.Fig.1e

Col-7			ckrc3		
91.393	103.276		87.064	116.2	98.43
118.795	93.911		120.945	93.057	121.189
106.725	116.435		103.88	179.664	162.474
111.865	96.71		112.916	358.331	354.21
119.066	100.067		356.87	53.042	99.707
82.013	99.106		358.94	95.292	326.343
95.97	107.727		116.837	358.61	111.912
102.754	122.052		92.472	98.448	86.041
86.137	90.64		87.503	110.557	85.695
108.263	89.619		2.721	94.888	98.565
83.817	88.736		90.767	108.134	76.859
103.489	84.592		99.938	122.034	91.878
112.814	92.72		83.824	100.103	179.84
103.998	91.21		74.32	83.623	96.865
93.837	91.409		64.059	101.947	91.662
94.289	90.021		81.067	109.64	120.36
81.304	92.34		355.33	97.508	123.366
108.521	94.185		86.486	88.521	
93.377	91.009		94.611	178.66	
91.619	94.479		106.385	118.937	
99.545	104.205		96.063	71.114	
94.505			65.265	83.809	
98.49			67.532	97.199	
86.737			82.208	111.644	
95.52			63.984	103.046	
101.769			100.274	102.441	
96.707			100.35	96.554	
83.528			102.848	118.922	
122.362			357.98	89.742	
94.844			98.162	116.354	

data.Fig.1g

Col-7	ckrc3
22.70	7.93
25.99	7.90
23.45	7.89
25.51	9.38
27.06	8.00
33.05	9.03
28.28	10.24
18.76	7.36
20.67	7.02
22.46	6.92
23.43	7.33
20.15	6.70
18.30	7.27
20.51	6.09
23.15	7.66
29.92	8.57
27.56	8.54
24.38	9.41
25.31	9.11
24.44	9.79
18.98	6.89
16.88	7.32
29.88	8.21
25.94	9.39
29.06	8.34
22.49	6.80
21.80	6.71
25.46	9.86
27.25	10.59
19.49	6.66
21.82	6.18

data.Fig.1j

MS	0.01uMIAA	0.1	0.1	0.5	1.0	MS
Col-7	Col-7	Col-7	Col-7	Col-7	Col-7	ckrc3
22.7	18.8	15	6.7	5.3	2.8	13.5
23.8	18.3	14.7	7.4	5.6	2.6	15.8
23.4	16	12.4	9.1	5	2.8	16.1
25.5	22.4	12.1	9.8	4.7	2.2	14
23.4	23.6	12.1	9.6	4.2	2.9	16.4
23.2	14.2	13.8	8.7	5.9	2.6	14.3
21.6	23.3	14.9	9.6	5.6	2.3	17.7
20.6	20.2	14.8	9	5.4	2.2	16.9
18.7	19.9	12.6	9.7	6	2.5	15.6
19.3	16.8	12.1	9	5.1	2.9	13.9
20.9	19.1	13.3	9.8	4.7	2.8	13.6
23	22.6	12.3	7.1	5.6	2.4	12.1
23.7	18.4	12.2	9.9	5.9	3.1	15.4
22.5	18.6	12.8	9.3	4.5	3	14.7
24.3	24.6	11.6	8.1	5.1	2.5	12.4
18.9	26.9	13	6.8		2.9	12.9
23.6	22.8	12.6	8.7			16.3
22.3	23.9	11.2	7.7			13.2
23.4	18	13.5	7.6			15.7
23.5	22.6	15.2	10.4			12.3
25.5	19.5	14.9	9.6			14.9
22.5	22.2	15	9.9			11.5
23.9	16.6	13.8	11			14
21.1	22.7	14.5	8.2			16.3
21	22	15.4	8.3			16.4
20.6	20.9	15.2	8.7			15.8
24.6	19.4	13.8	6.7			15.6
22.7	20.6	14.7	8.6			13.1
23.4	18.5	13.1	6.5			12.2
23.5	20.2	11.7	7.7			14.5
	23.5	12.3	8.1			
	18.6	14.5	7			
	23.3	12.3	8.1			
	19.2	15.8	9.4			
	20.8		7.1			
	22.3		8.9			
	17.6		8.4			
	19.1		9.7			
	19.4		11.6			
	19.9		10.2			
			9.8			
			8.3			
			8.3			

0.01uMIAA	0.1	0.1	0.5	1.0
ckrc3	ckrc3	ckrc3	ckrc3	ckrc3
14	14.4	8.9	5.2	2.6
15.1	17.7	8.2	5	3.9
22.2	16.6	11	4.3	3.8
20.6	17	10.3	5.9	3
22.5	14.6	10.5	6.6	2.1
19.4	14	10.5	5.7	2.7
21.4	14.4	11.2	4.8	2.9
21.2	19	11.1	5.1	3
16.9	16.6	11.3	5.3	2.1
19.1	15.8	11.8	5.6	2.4
21.1	15.8	9.2	6.1	3
22.5	15.1	10.8	5.3	2.3
21.7	16.5	9.7	5.9	2.2
18.4	14.1	11.5	5.5	2.7
17.7	15.7	8	5.7	2.9
18.2	14.1	11.5		2.6
21.3	17.2	11.3		
22.8	14.2	9.5		
19.3	14.1	8.6		
20.7	16.3	8.9		
21.4	15.8	11.7		
24.3	14.8	11.1		
24.3	16.6	11.3		
22.6	15.9	9.9		
24.6	14.8	8		
19.8	16.9	8.1		
17.4	13.9	11.7		
19.7	15.3	10.9		
14.9	15	9		
18	14.7	7.9		
22.2	15.5	9.7		
18.3	16	10		
23	15.6	9.1		
17.6	16	11.6		
21	15.3	11.6		
17.1		8.2		
		9.9		
		11.5		
		11		
		10.2		
		10.3		

data.Fig.2d

Col-7	ckrc3	Col-0	nbc-1	ckrc3 nbc-1
19.9	9.3	19.7	12.2	12.7
22.6	10.4	20.7	12.3	14.1
21.6	13.0	19.1	13.1	13.2
21.3	11.4	19.1	12.4	13.6
20.3	10.1	20.6	13.1	11.6
20.0	9.3	19.1	14.0	12.6
18.5	13.8	20.5	14.7	11.0
19.5	9.8	17.8	13.4	11.4
18.9	11.3	19.0	12.5	12.6
19.2	10.0	18.8	13.5	12.5
20.7	11.5	21.8	12.3	12.2
20.0	13.9	23.6	12.7	12.3
20.1	12.0	19.1	13.4	11.1
18.5	11.2	19.8	14.5	10.6
21.8	12.9	23.6	15.5	10.2
20.1	12.1	18.1	13.8	15.6
19.0	11.0	22.4	10.8	14.6
18.1	10.8	20.2	14.9	12.7
19.5	14.4	19.5	15.9	10.7
19.0	15.0	19.6	15.9	
18.3	10.3	20.4	15.4	
19.0	12.8	22.3	15.3	
	11.6	18.9	14.3	
	11.3	19.4	13.3	
		22.6	13.7	
		22.9	14.0	
		19.5		

data.Fig.2e

Col-7	ckrc3	Col-0	nbc-1	ckrc3 nbc-1
0.923	0.836	0.975	0.738	0.808
0.954	0.698	0.910	0.846	0.671
0.926	0.852	0.960	0.923	0.792
0.927	0.838	0.924	0.834	0.816
0.986	0.792	1.000	0.851	0.858
1.000	0.776	0.962	0.838	0.886
0.951	0.736	0.926	0.846	0.773
0.988	0.624	0.978	0.794	0.757
0.892	0.693	1.000	0.840	0.805
0.978	0.800	0.972	0.921	0.728
0.975	0.579	0.981	0.835	0.718
1.000	0.674	0.955	0.758	0.788
0.950	0.776	0.923	0.828	
0.959	0.699	0.992	0.850	
0.968	0.753	0.981	0.870	
0.995	0.827	0.970		
0.999	0.739	1.000		
0.985	0.673	0.995		
0.966	0.770	0.976		
0.993	0.736	0.991		
1.000	0.809	0.987		
	0.773	1.000		
	0.835	0.986		
	0.772	0.954		
	0.807	0.999		

data.Fig.2g

Col-0			nbc-1	
102.392	64.332		107.218	90.581
119.82	93.797		136.312	125.865
110.375	105.825		118.352	102.51
100.188	93.4		79.011	117.128
99.774	89.927		126.393	97.904
99.56	104.567		121.567	113.63
96.566	114.923		120.094	109.57
99.326	86.611		88.761	142.502
116.154	81.87		137.956	125.874
123.111	74.152		100.883	
85.657	102.579		122.183	
109.396	101.449		132.008	
104.004	85.987		117.177	
98.015	84.336		81.503	
112.099	100.998		175.701	
93.559	95.16		129.145	
73.964	99.29		116.208	
102.81	76.098		133.611	
99.604	111.863		111.006	
93.857	92.387		126.038	
113.153	87.99		119.335	
87.917	88.348		120.197	
102.916	109.127		126.342	
95.123	107.743		104.585	
79.923	96.319		107.722	
77.455			111.371	
84.289			113.727	
84.109			131.082	
81.257			98.89	
102.375			125.562	

data.Fig.2i

MS	0.01uMIAA	0.1	0.1	0.5	1.0	MS
Col-7	Col-7	Col-7	Col-7	Col-7	Col-7	ckrc3
22.7	18.8	15.0	6.7	5.3	2.8	13.5
23.8	18.3	14.7	7.4	5.6	2.6	15.8
23.4	16	12.4	9.1	5.0	2.8	16.1
25.5	22.4	12.1	9.8	4.7	2.2	14.0
23.4	23.6	12.1	9.6	4.2	2.9	16.4
23.2	14.2	13.8	8.7	5.9	2.6	14.3
21.6	23.3	14.9	9.6	5.6	2.3	17.7
20.6	20.2	14.8	9.0	5.4	2.2	16.9
18.7	19.9	12.6	9.7	6.0	2.5	15.6
19.3	16.8	12.1	9.0	5.1	2.9	13.9
20.9	19.1	13.3	9.8	4.7	2.8	13.6
23	22.6	12.3	7.1	5.6	2.4	12.1
23.7	18.4	12.2	9.9	5.9	3.1	15.4
22.5	18.6	12.8	9.3	4.5	3.0	14.7
24.3	24.6	11.6	8.1	5.1	2.5	12.4
18.9	26.9	13.0	6.8		2.9	12.9
23.6	22.8	12.6	8.7			16.3
22.3	23.9	11.2	7.7			13.2
23.4	18	13.5	7.6			15.7
23.5	22.6	15.2	10.4			12.3
25.5	19.5	14.9	9.6			14.9
22.5	22.2	15.0	9.9			11.5
23.9	16.6	13.8	11.0			14.0
21.1	22.7	14.5	8.2			16.3
21	22	15.4	8.3			16.4
20.6	20.9	15.2	8.7			15.8
24.6	19.4	13.8	6.7			15.6
22.7	20.6	14.7	8.6			13.1
23.4	18.5	13.1	6.5			12.2
23.5	20.2	11.7	7.7			14.5
	23.5	12.3	8.1			
	18.6	14.5	7.0			
	23.3	12.3	8.1			
	19.2	15.8	9.4			
	20.8		7.1			
	22.3		8.9			
	17.6		8.4			
	19.1		9.7			
	19.4		11.6			
	19.9		10.2			
			9.8			
			8.3			
			8.3			

0.01uMIAA ckrc3	0.1 ckrc3	0.1 ckrc3	0.5 ckrc3	1.0 ckrc3	MS ckrc3 nbc-1	0.01uMIAA ckrc3 nbc-1
14.0	14.4	8.9	5.2	2.6	15.5	18.2
15.1	17.7	8.2	5.0	3.9	16.1	24.2
22.2	16.6	11.0	4.3	3.8	13.1	18.3
20.6	17.0	10.3	5.9	3.0	11.8	21.3
22.5	14.6	10.5	6.6	2.1	13.5	17.7
19.4	14.0	10.5	5.7	2.7	14.4	21.3
21.4	14.4	11.2	4.8	2.9	15.8	22.0
21.2	19.0	11.1	5.1	3.0	14.6	20.7
16.9	16.6	11.3	5.3	2.1	17.2	17.9
19.1	15.8	11.8	5.6	2.4	18.1	18.8
21.1	15.8	9.2	6.1	3.0	15.8	19.7
22.5	15.1	10.8	5.3	2.3	15.9	19.7
21.7	16.5	9.7	5.9	2.2	15.4	23.7
18.4	14.1	11.5	5.5	2.7	18.8	23.8
17.7	15.7	8.0	5.7	2.9	19.0	19.4
18.2	14.1	11.5		2.6	16.8	21.3
21.3	17.2	11.3			17.9	17.3
22.8	14.2	9.5			15.5	18.3
19.3	14.1	8.6			16.7	23.1
20.7	16.3	8.9			16.3	17.8
21.4	15.8	11.7			17.3	21.9
24.3	14.8	11.1			13.2	19.7
24.3	16.6	11.3			17.4	23.1
22.6	15.9	9.9			15.9	19.2
24.6	14.8	8.0			16.7	21.1
19.8	16.9	8.1			17.3	22.9
17.4	13.9	11.7			18.8	22.3
19.7	15.3	10.9			14.9	19.9
14.9	15.0	9.0			13.7	18.8
18.0	14.7	7.9			15.4	20.9
22.2	15.5	9.7				17.5
18.3	16.0	10.0				19.7
23.0	15.6	9.1				19.7
17.6	16.0	11.6				19.5
21.0	15.3	11.6				19.7
17.1		8.2				18.2
		9.9				23.1
		11.5				21.3
		11.0				21.3
		10.2				21.7
		10.3				22.3
						19.3

0.1	0.1	0.5	1.0	MS	0.01uMIAA	0.1
ckrc3 nbc-1	ckrc3 nbc-1	ckrc3 nbc-1	ckrc3 nbc-1	Col-0	Col-0	Col-0
17.4	9.1	5.6	2.3	21.3	17.1	16.2
17.2	9.6	6.6	2.4	21.1	16.8	11.3
18.0	9.8	6.3	2.5	22.7	18.6	12.8
14.2	10.1	6.4	2.6	23.4	21.6	13.9
16.3	10.7	5.7	2.3	21.4	20.5	12.9
17.1	9.8	5.7	2.4	24.0	19.4	12.8
18.8	9.7	6.4	2.8	21.7	19.7	16.1
15.0	11.5	6.9	2.2	21.1	18.5	13.1
13.7	12.3	5.7	2.2	23.6	19.9	14.8
18.7	10.2	5.8	2.3	25.0	19.3	14.2
15.7	9.5	4.8	2.2	22.4	19.0	12.6
14.4	12.2	5.4	3.0	21.8	22.0	15.0
19.0	11.0	5.5	2.2	21.2	23.2	13.7
17.7	10.7	5.8	2.3	20.9	18.1	12.5
15.5	11.7	5.5	2.7	20.1	24.3	13.3
15.0	10.6		2.9	21.0	24.6	14.5
15.1	13.4			22.1	20.6	16.4
14.5	9.9			20.3	21.2	12.8
14.4	12.3			23.3	22.9	16.3
18.5	10.6			22.9	23.0	14.7
17.1	10.3			23.6	19.9	16.1
17.4	13.5			20.2	20.9	15.7
16.3	14.0			22.7	22.5	14.3
15.1	10.3			21.8	22.7	12.7
17.2	9.7			21.9	19.2	12.6
16.9	11.0			23.4	18.0	15.6
14.2	8.4			24.9	19.4	13.3
15.5	9.4			22.7	20.7	13.7
16.9	9.7			22.3	19.3	11.5
18.9	9.3			20.0	20.3	12.3
14.3	11.1				22.2	13.0
15.4	14.0				18.8	14.6
14.6	12.5				20.2	13.4
15.4	12.4				19.7	12.7
19.2	14.8				18.4	15.5
14.7	12.5				20.6	
	13.3				21.1	
	12.2				19.1	
	12.7					
	14.9					

0.1	0.5	1.0	MS	0.01uMIAA	0.1	0.1	0.5
Col-0	Col-0	Col-0	nbc-1	nbc-1	nbc-1	nbc-1	nbc-1
9.2	5.4	3.3	15.9	22.5	15.9	10.9	5.1
9.4	6.0	2.6	13.8	13.5	15.2	12.6	4.9
8.0	5.4	2.2	15.8	21.8	16.2	11.2	5.3
9.6	5.2	2.0	14.8	18.1	16.3	9.0	6.4
9.3	4.5	2.2	16.9	21.8	17.3	11.8	5.6
9.8	5.8	2.7	13.3	22.8	15.8	11.4	6.2
8.9	5.1	2.3	15.5	20.3	12.9	10.0	5.9
10.6	5.7	2.9	15.7	18.0	16.7	10.3	5.2
9.1	5.3	3.5	15.1	18.1	14.4	9.3	6.2
7.0	5.2	2.7	13.2	19.8	18.6	11.1	6.1
10.7	5.3	3.0	13.5	24.9	13.0	12.4	5.3
6.8	4.8	2.2	14.9	23.8	15.1	9.5	5.4
7.2	4.9	2.1	15.0	23.1	17.1	12.3	5.1
9.2	4.6	2.6	14.0	20.0	15.8	9.6	4.5
9.9	5.2	2.0	16.9	21.6	15.6	10.1	5.4
10.0		2.4	14.4	17.5	17.5	11.3	
8.8			15.8	17.8	15.6	10.1	
10.0			15.0	23.8	14.6	12.5	
9.7			16.6	18.1	14.9	10.7	
8.9			15.9	21.8	17.9	11.4	
10.6			16.8	19.1	14.6	9.5	
8.5			17.0	23.2	14.3	11.9	
10.0			15.3	19.2	17.9	9.8	
9.7			16.7	21.8	14.3	11.0	
10.0			15.5	19.3	14.9	9.6	
8.0			15.4	21.5	14.5	12.5	
10.2			15.9	15.3	14.9	9.3	
9.4			13.6	17.7	16.5	12.3	
8.5			17.1	20.4	15.9	10.4	
9.9			16.5	17.7	15.6	9.2	
9.3				24.2	15.1	10.9	
10.5				18.0	15.7	9.6	
9.5				17.2	14.6	11.6	
10.5				17.3	14.8	11.6	
9.2				18.3	16.3	10.5	
11.5				21.9	14.2		
10.0				21.7			
9.4				21.1			
9.8							

1.0
nbc-1
2.9
2.8
2.8
2.1
2.4
2.7
2.2
2.7
2.6
2.9
2.4
2.1
3.9
2.1
2.8
2.7

data.Fig.2j

Col-7	ckrc3	Col-0	nbc-1	ckrc3 nbc-1
22.70	7.90	23.30	9.30	7.50
26.00	7.60	27.70	10.30	6.00
21.50	4.96	22.30	9.60	11.65
23.43	8.51	18.38	5.74	7.57
13.30	9.25	16.13	9.96	8.49
27.57	9.02	27.59	8.79	9.87
27.18	9.22	27.97	9.68	8.96
24.84	7.84	25.24	9.60	9.41
27.71	9.19	29.99	9.75	8.31
27.25	8.28	27.02	9.32	8.24
27.38	11.67	28.95	9.40	4.36
23.37	5.05	14.22	7.93	6.48
14.53	6.63	12.66	6.87	8.30
29.23	8.44	28.12	9.17	7.62
17.73	6.21	20.32	7.53	7.06
18.23	6.15	23.61	6.73	7.17
20.44	7.90	21.95	7.62	7.47
20.58	6.98	19.92	7.84	7.83
23.30	6.55	20.55	7.69	7.73
19.58	6.86	27.57	6.91	9.46
31.62	10.15	32.96	11.87	7.00
15.38	6.42	21.95	7.98	6.53
21.00	5.92	24.12	6.86	6.09
19.56	7.68	23.24	8.11	6.77
21.39	6.16	18.73	8.58	7.37
28.19	8.34	26.28	10.60	9.63
28.50	9.03	25.76	9.02	9.09
27.26	7.67	29.81	6.68	8.75
23.21	7.19	20.66	9.84	6.65
31.43	10.22	32.53	9.25	10.93
34.04	10.57	32.53	11.17	8.01

data.Fig.3b

Col-7	ckrc3nbc-1	#1	#13	#3	#7
1.692	1.48	3.07	3.23	1.46	1.442
1.514	1.38	3.46	3.28	1.78	1.656
1.904	1.52	3.5	2.91	1.77	1.742
1.291	1.61	4.44	3.41	1.58	1.604
1.709	1.58	3.92	2.68	1.53	1.673
1.565	1.6	3.71	3.7	1.96	1.236
1.732	1.58	4.31	3.26	1.71	1.591
1.793	1.21	3.35	3.9	1.86	1.623
1.379	1.59	3.23	3.04	1.56	1.471
1.379	1.49	3.16	3.61	1.92	1.601
1.441	1.32	3.54	2.98	1.73	1.529
1.639	1.29	4.22	3.16	1.56	1.992

data.Fig.3c

Col-7	#1	#13	#3	#7
1.153912	1.619222	5.104505	16.409650	16.364800
1.127433	1.559694	6.043160	10.822030	11.595630
0.718655	1.652293	4.916322	9.420637	13.063970
1.203606	6.350016	6.641935	17.128730	13.023590
0.982270	4.448136	5.399297	13.620500	8.403647
0.814124	3.230031	7.271116	33.616560	10.558850
1.105684	5.885830	9.818208	17.223050	11.172920
1.002316	6.785646	8.039442	13.008500	12.282070
0.893214	6.684787	6.640952	13.372060	12.609840

data.Fig.4b

yuc8	#3	#5	#7	#52	#60	#67
1.614	2.771	3.243	2.458	1.898	1.554	1.882
1.756	3.584	3.115	2.240	1.762	1.463	1.936
1.421	4.204	2.820	2.675	1.882	1.472	1.876
2.026	3.633	2.602	2.314	1.681	1.689	1.472
1.534	4.111	2.557	2.626	1.941	1.642	1.738
1.614	4.137	3.264	3.268	1.936	1.594	1.876
1.594	3.883	2.853	2.466	1.588	1.732	1.534
1.882	4.187	2.902	2.964	1.291	1.766	1.331
1.369	3.596	3.294	3.010	1.646	1.742	1.443
1.621	3.904	3.357	2.463	1.646	1.534	1.744
1.646	3.105	2.922	2.282	1.837	1.331	1.413
1.534	4.372	2.776	2.972		1.443	1.447

data.Fig.4c

Col-0	yuc8	#3	#5	#7	#52	#60	#67
0.890	0.619	0.924	0.955	0.928	0.547	0.526	0.547
0.991	0.339	0.988	1.000	0.955	0.608	0.553	0.609
0.958	0.592	0.997	0.890	0.995	0.471	0.578	0.587
0.923	0.670	1.000	0.998	0.984	0.702	0.642	0.554
0.990	0.592	0.946	0.922	0.977	0.707	0.591	0.462
0.962	0.624	0.981	0.963	0.949	0.550	0.587	0.621
0.925	0.700	0.957	0.948	0.920	0.541	0.623	0.402
0.911	0.505	0.947	0.957	1.000	0.533	0.531	0.430
0.998	0.464	0.892	0.935	1.000	0.695	0.503	0.533
0.991	0.593	0.995	0.983	0.966	0.629	0.509	0.554
0.995	0.586	0.920	0.993	0.983	0.603	0.675	0.639
0.955	0.543	0.865	0.958	0.973	0.506	0.557	0.624
0.980	0.458	1.006	0.966	1.000	0.547	0.557	0.559
0.974	0.649	0.952	0.976	0.995	0.630	0.669	0.503
0.998	0.621	0.955	1.000	0.962	0.645	0.592	0.371
0.967	0.431	0.917	0.958	0.923	0.547	0.671	0.459
0.976	0.583	1.000	0.994	0.913	0.590	0.592	0.681
0.994	0.605	0.995	0.898	0.993	0.649	0.671	0.505
0.997	0.585	0.910	0.993	0.905	0.507	0.822	0.734
1.000	0.824	0.961	0.939	0.979	0.604	0.417	0.590
0.996	0.540	1.000	0.994	0.956	0.675	0.591	0.715
0.962	0.468	0.998	0.952	0.999	0.421	0.455	0.593
0.946	0.643	0.742	0.960	0.987	0.387	0.732	0.505
0.990	0.587		0.910	0.993		0.537	0.700
0.982	0.622		0.998	0.966		0.417	0.705
0.991	0.507		0.983	0.997		0.700	0.651
1.000	0.401		0.900			0.464	0.457
	0.423		0.962			0.504	0.641
						0.538	0.640
						0.715	0.639

data.Fig.4d

yuc8	col-0	#3	#5	#7	#52	#60	#67
0.000979	0.980274	3.262902	6.025627	11.81425	4.953325	2.573611	15.31111
0.000857	1.265689	3.632911	3.957088	12.84004	3.312255	2.660887	19.09498
0.001022	0.754036	2.941887	6.536465	13.016	4.434822	3.097236	12.11781
0.000637	1.011447	5.30763	2.513962	4.571687	2.679016	5.986233	10.21114
0.000719	1.091387	4.632846	3.542485	3.626319	3.480093	6.253357	12.21213
0.000614	0.897166	3.437988	4.055381	2.523498	3.678613	6.057206	11.94648
0.000662	1.203606	2.15739	3.841865	3.581343	2.992019	3.690261	11.96263
0.000533	0.98227	1.950516	2.862956	3.369035	2.841572	4.842012	15.96732
0.000982	0.814124	1.932367	2.495242	3.297854	2.615863	4.463446	17.06945

data.Fig.S1g

Col-7	0-30°	1.00
ckrc3	0-30°	0.12
	31-60°	0.29
	61-90°	0.38
	91-120°	0.14
	121-150°	0.06
	151-180°	0.01

data.Fig.S1h

col-7			ckrc3		
1	2	3	1	2	3
10.5	11.2	5.8	8.8	7.2	8.1
10.5	10.1	8.0	10.4	4.9	5.5
9.1	10.9	7.8	9.1	4.5	6.5
9.2	10.5	8.2	4.9	4.9	5.5
11.1	10.4	7.3	7.6	4.5	5.6
9.5	7.4	10.1	9.1	4.1	5.4
9.5	9.8	5.4	6.3	5.1	4.8
10.1	5.9	8.8	7.7	4.1	4.6
8.5	7.4	7.5	8.4	3.9	5.5
13.2	8.4	8.7	4.4	2.4	4.8
6.6	8.1	8.2	5.5	3.4	3.3
7.0	5.4	5.9	8.1	4.5	6.5
12.6	9.5	7.4	5.2	5.8	4.3
9.9	10.0	10.1	5.4	5.1	5.1
7.4	7.8	6.7	4.6	4.0	3.1
7.1	8.0	6.7	8.5	3.8	5.3
8.5	5.9	9.7	6.0	3.3	4.4
10.6	8.8	6.5	7.9	3.1	8.8
8.5	12.1	9.2	6.3	4.7	5.0
8.6	9.9	9.3	5.6	4.8	8.7
7.1	8.8	7.5	7.2	4.3	8.5
9.5	9.8	7.4	7.0	5.6	5.7
9.9	7.0	9.1	6.0	5.6	8.9
9.2	7.5	10.5	5.2	4.7	4.1
17.5	5.8	9.0	6.0	6.3	6.6
12.4	8.5	9.2	5.2	7.0	4.7
10.8	7.6	8.6	5.8	4.7	6.1
12.4	7.9	6.2	4.9	2.9	6.7
11.3	9.8	7.6	3.7	5.3	4.3
10.7	6.8	8.8	6.4	5.5	6.8
13.5	6.2	10.0	6.0	3.7	8.0
7.7	6.2	7.8	7.0	4.0	5.8
10.7	6.2	8.0	6.5	7.1	5.4
8.4	6.1	5.9	4.9	6.0	5.1
8.3	8.2	8.8	4.0	5.8	2.5
8.9	9.7	12.1	5.6	5.0	4.6
9.1	7.7	9.9	6.3	4.2	3.3
11.1	9.5	8.8	3.8	3.7	3.4
12.0	5.9	9.8	4.5	5.3	4.3
12.0	5.9	7.0	4.6	3.7	3.8
13.2	9.8	7.5	3.2	3.7	4.4
11.5	8.9	5.8	3.1	6.5	5.2
9.3	8.9	8.5	3.9	6.9	4.9
8.1	12.6	7.6	5.5	6.9	4.9
10.8	11.6	7.9	5.3	3.7	5.1
6.1	8.4	9.8	4.1	4.8	6.8
7.0	8.9	6.8	3.1	7.1	4.7
8.7	8.9	6.2	3.1	4.7	

11.1	10.8	6.2
12.7	9.5	6.2
10.1	9.0	6.1
7.8	10.2	8.2
7.8	10.3	9.7
7.4	5.7	7.7
8.2	9.4	10.9
10.0	7.8	8.4
9.6	11.5	6.8
10.5	5.5	8.3
6.5	11.4	6.4
7.3	8.0	6.1
8.8	10.6	6.7
8.9	6.7	9.2
4.6	7.4	8.3
9.6	9.8	6.5
7.3	9.2	9.7
4.5	8.6	9.4
6.8	6.4	9.8
6.6	5.5	7.0
7.4	9.2	8.9
6.8	5.8	10.8
10.4	7.4	6.4
8.8	6.8	11.1
7.2	7.9	7.5
5.9	7.2	9.8
6.9	7.9	5.9
6.0	10.6	7.4
7.5	10.1	8.4
7.2	7.1	8.1
8.3	10.7	5.4
6.9	8.5	9.5
8.2	8.6	10.5
5.9	7.9	10.4
11.2	7.1	7.4
10.1	7.3	9.4
10.9	9.5	12.9

data.Fig.S1i

col-7			ckrc3		
1	2	3	1	2	3
394.6	244.4	282.5	129.6	214.9	192.8
309.4	290.5	286.7	126.9	189.7	190.9
308.1	336.9	300.9	163.9	187.3	180.1
332.8	280.3	354.4	190.6	172.4	180.1
334.3	329.5	311.4	144.6	182.0	183.1
289.9	346.7	344.9	181.0	174.2	146.0
362.4	286.5	314.6	173.3	118.4	181.7
290.8	244.4	316.7	156.2	166.4	182.5
330.1	290.5	282.4	164.9	185.2	216.6
312.8	359.9	255.2	155.2	181.8	192.4
320.3	342.6	311.1	204.8	168.3	113.3
297.2	328.1	365.7	172.6	187.9	129.0
285.3	330.0	361.6	167.3	172.3	154.0
240.1	311.6	270.4	175.7	142.1	210.9
279.6	378.0	281.1	149.9	132.9	190.2
336.9	362.4	346.7	164.5	125.4	201.1
280.3	296.2	286.5	165.2	171.6	182.7
329.5			210.3	128.2	183.3
			146.3	136.9	157.1
			174.4	115.6	186.3
			237.3	249.6	197.3
			232.9	213.8	163.8
			182.8	193.9	163.9
			208.9	192.1	165.8
			193.1	148.7	175.9
			183.1	98.5	169.4
			120.0	182.1	154.6
				201.3	143.1

data.Fig.S2

MS						0.1uM tZ		
Col-7	ckrc3	Col-0	nbc-1	ckrc3 nbc-1		Col-7	ckrc3	Col-0
91.4	87.1	102.4	107.2	103.1		140.8	118.6	143.7
118.8	120.9	119.8	136.3	111.6		91.9	360.0	136.9
106.7	103.9	110.4	118.4	110.4		95.9	175.5	126.4
111.9	112.9	100.2	79.0	88.4		129.8	163.4	115.7
119.1	360.0	99.8	126.4	126.5		147.9	360.0	147.7
82.0	360.0	99.6	121.6	118.1		157.7	142.4	118.7
96.0	116.8	96.6	120.1	90.0		88.4	169.4	114.2
102.8	92.5	99.3	88.8	110.0		139.0	130.2	92.7
86.1	87.5	116.2	138.0	93.1		125.2	96.8	158.4
108.3	0.0	123.1	100.9	111.8		110.6	169.0	160.5
83.8	90.8	85.7	122.2	102.6		116.0	174.5	160.5
103.5	99.9	109.4	132.0	110.5		126.6	176.9	134.4
112.8	83.8	104.0	117.2	117.0		128.1	316.2	148.1
104.0	74.3	98.0	81.5	130.0		136.9	103.8	94.4
93.8	64.1	112.1	175.7	96.9		130.5	267.6	114.0
94.3	81.1	93.6	129.1	63.4		141.8	157.8	75.5
81.3	360.0	74.0	116.2	114.6		101.9	122.7	116.2
108.5	86.5	102.8	133.6	99.9		122.6	131.1	138.7
93.4	94.6	99.6	111.0	104.5		113.4	111.3	123.7
91.6	106.4	93.9	126.0	110.8		134.0	289.7	106.3
99.5	96.1	113.2	119.3	109.6		93.0	167.3	156.2
94.5	65.3	87.9	120.2	89.2		81.0	360.0	143.7
98.5	67.5	102.9	126.3	110.0		105.7	85.9	161.2
86.7	82.2	95.1	104.6	93.3		128.4	85.9	135.4
95.5	64.0	79.9	107.7	92.2		116.6	95.2	106.8
101.8	100.3	77.5	111.4	104.1		100.0	107.0	132.9
96.7	100.4	84.3	113.7	111.5		78.6	129.4	103.4
83.5	102.8	84.1	131.1	100.5		111.8	178.2	106.5
122.4	360.0	81.3	98.9	124.5		88.4	154.2	120.2
94.8	98.2	102.4	125.6	93.1		130.8	160.3	135.3
103.3	116.2	64.3	90.6	76.6		91.4	175.9	89.9
93.9	93.1	93.8	125.9	97.7		87.1	163.4	123.6
116.4	180.0	105.8	102.5	101.7		71.5	167.5	126.0
96.7	360.0	93.4	117.1	114.6		149.4	171.9	143.5
100.1	53.0	89.9	97.9	85.5		63.1	152.3	132.3
99.1	95.3	104.6	113.6	106.2		78.1	168.1	120.4
107.7	360.0	114.9	109.6	111.3		131.9	136.6	84.9
122.1	98.4	86.6	142.5	101.9		54.2	148.9	132.4
90.0	110.6	81.9	125.9	119.5		138.9	134.6	121.8
89.6	94.9	74.2		96.0		100.8	89.3	107.7
88.7	108.1	102.6		98.9		98.4	131.8	116.6
84.6	122.0	101.4		103.8		104.6	161.8	67.7
92.7	100.1	86.0		105.0		124.3	152.0	75.9
90.0	83.6	84.3		123.2		67.3	141.0	78.5
91.4	101.9	101.0		89.0		114.8	178.5	128.1
90.0	109.6	95.2		90.0		163.2	112.3	95.0
94.2	97.5	99.3		96.3		52.3	127.1	109.7
91.0	88.5	76.1		87.6		113.9	65.5	66.8

94.5	180.0	111.9		86.4		91.7	177.5	88.5
104.2	118.9	92.4		128.9		107.6	130.5	67.8
	71.1	88.0		97.9		94.8	95.5	113.0
	83.8	88.3		132.2		125.5	113.8	111.6
	97.2	109.1		114.3			149.4	115.8
	111.6	107.7		114.2			103.0	110.7
	103.0	96.3		90.8			360.0	112.8
	102.4			102.4			334.6	94.1
	96.6			86.1			139.0	140.7
	118.9			98.1			357.4	
	89.7			86.1			157.6	
	116.4			92.3			140.0	
	98.4						110.0	
	121.2						142.8	
	162.5						136.5	
	360.0							
	99.7							
	326.3							
	111.9							
	86.0							
	85.7							
	98.6							
	76.9							
	91.9							
	180.0							
	96.9							
	91.7							
	120.4							
	123.4							

		0.01uM IAA				
nbc-1	ckrc3 nbc-1	Col-7	ckrc3	Col-0	nbc-1	ckrc3 nbc-1
160.8	85.1	101.8	99.3	98.6	112.1	104.3
139.2	106.4	98.4	92.1	86.1	110.1	95.2
154.9	167.5	88.9	98.1	107.6	104.5	105.6
166.9	360.0	84.6	101.9	119.1	105.8	111.1
58.1	180.0	93.1	105.3	86.2	121.1	96.3
167.3	360.0	94.2	90.0	101.7	94.5	97.7
152.7	175.4	96.7	110.9	126.0	109.9	128.5
132.1	143.1	84.3	92.2	98.4	95.6	114.9
113.9	129.7	102.1	86.8	100.8	112.7	108.9
360.0	170.7	99.5	84.4	97.1	125.7	129.5
164.4	150.8	92.7	113.6	117.1	112.2	96.3
177.6	149.5	100.0	81.7	64.5	103.2	124.5
138.7	360.0	103.3	125.3	115.0	94.5	88.0
112.1	107.7	97.1	114.4	129.6	108.4	107.3
124.2	178.4	119.4	81.0	79.3	116.2	87.1
127.7	158.5	105.0	90.0	88.4	94.1	91.2
180.0	148.5	122.6	93.4	98.3	123.2	97.2
175.4	300.0	80.1	96.0	99.6	124.2	93.9
171.5	155.0	105.6	109.6	118.9	118.0	116.6
167.4	149.5	125.4	107.9	113.1	111.9	72.4
151.3	88.9	94.2	124.4	115.2	118.2	107.2
154.4	121.2	105.6	109.1	85.5	115.7	129.2
91.7	114.0	81.0	116.7	97.8	104.2	115.1
143.1	138.4	91.9	84.2	90.0	108.1	114.2
143.4	318.9	88.3	91.5	86.9	102.1	103.0
126.2	127.0	96.0	93.2	90.6	101.3	91.6
169.4	177.3	90.0	86.6	108.6	105.8	100.1
142.8	360.0	87.1	97.8	88.2	97.8	95.6
149.8	165.1	132.4	100.6	83.5	95.3	99.0
178.8	84.0	110.1	78.4	90.0	104.6	96.5
143.4	112.5	114.4	75.1	84.0	80.4	80.8
174.2	360.0	92.8	110.0	93.9	93.0	107.3
163.2	133.9	79.7	76.7	95.1	97.6	118.8
147.8	340.7	88.6	95.4	84.5	76.4	84.8
118.5	319.9	94.5	77.3	96.6	89.7	121.9
173.2	85.1	120.7	109.0	88.4	80.5	116.2
158.7	137.9	96.7	81.6	83.2	100.8	94.7
161.4	108.8	101.0	72.4	91.6	81.5	109.4
102.7	335.2	90.4	83.5	106.7	101.5	97.1
109.4	360.0	93.4	85.1	132.4	80.8	78.3
98.6	360.0	81.2	97.9	70.3	83.1	111.9
114.6	360.0	87.4	88.7	108.3	93.8	89.1
102.1	72.4	88.0	86.2	96.8		101.3
103.6	292.4	92.5	88.9	85.1		125.1
108.0	177.9	54.9	116.6	108.1		90.0
168.3	130.1	82.9	92.3	92.3		114.8
155.1	360.0	109.0	77.5	113.5		115.8
118.6	307.5	91.3	106.3	90.0		116.5

360.0	127.8	92.1	109.6	86.0
360.0	73.5	96.0	95.1	86.4
118.1	273.6	102.8	94.9	92.5
172.5	133.7	106.7	99.2	110.6
136.6	133.6	96.5	86.4	106.1
	116.6		99.5	113.8
	360.0		78.3	78.6
	159.2		101.0	103.2
	180.0		84.9	84.5
	360.0		103.1	103.1
	101.8		105.0	106.1
	110.0		95.4	114.5
	104.7			121.3
	252.4			114.6
	136.5			
	177.9			

data.Fig.S3f

Col-7	ckrc3	ckrc3-2	pCKRC3:CKRC3/ckrc3
19.0	11.0	11.1	16.3
18.1	12.3	15.5	18.2
19.1	12.4	11.7	16.8
15.7	10.3	11.3	17.1
15.2	9.6	14.4	14.2
16.9	13.3	12.1	16.5
17.7	9.3	13.1	15.7
18.2	11.6	10.3	16.9
18.6	10.6	10.5	17.7
19.1	12.4	11.7	17.2
18.0	10.3	11.3	17.1
19.7	11.0	11.1	16.3
19.8	11.4	12.2	17.4
23.0	12.2	13.8	17.6
15.9	12.2	9.6	18.5
16.8	12.0	10.5	16.8
20.8	11.3	11.4	15.9
19.3	14.2	10.6	17.5
18.3	15.1	8.9	19.6
18.5	12.7	10.4	16.1
22.1	12.1	11.5	19.3
18.5	11.9	11.3	17.9
17.8	13.7	12.4	18.1

data.Fig.S3g

Col-7	ckrc3	ckrc3-2	pCKRC3:CKRC3/ckrc3
0.975	0.737	0.882	0.975
0.917	0.571	0.757	0.970
0.870	0.713	0.822	0.922
0.909	0.750	0.740	0.952
0.965	0.727	0.669	0.832
0.996	0.769	0.765	0.987
0.900	0.667	0.692	1.000
0.947	0.776	0.756	0.963
0.994	0.763	0.813	0.984
0.917	0.771	0.867	0.898
0.917	0.692	0.750	0.956
0.986	0.749	0.771	0.989
1.000	0.857	0.769	0.993
0.952	0.692	0.744	0.947
0.996	0.733	0.779	0.965
0.965	0.846	0.690	0.910
0.964	0.786	0.765	0.944
1.000	0.741		1.000
0.997	0.757		
0.987	0.756		
0.960	0.757		
	0.833		
	0.803		
	0.765		

data.Fig.S5a

rosette leaf	cauline leaf	stem	root	flower	silique
0.963349	0.457804	0.221615	0.523453	0.568854	0.364199
1.011244	0.518638	0.221615	0.950086	0.588915	0.377043
1.025360	0.651935	0.214066	0.362520	0.597136	0.439155
0.963312	0.556321	0.198532	0.882542	0.456321	0.356321
1.012221	0.412362	0.220362	0.980632	0.495136	0.286302
1.024467	0.663212	0.250026	0.910652	0.403694	0.327985
0.893212	0.703268	0.193563	0.952103	0.586321	0.440044
0.936218	0.666213	0.183258	0.966231	0.602360	0.458964
1.170570	0.429832	0.239812	0.956975	0.690001	0.400569

data.Fig.S6d

Col-7	ckrc3	Col-0	nbc-1	ckrc3 nbc-1
313	170	319	187	174
325	168	317	177	164
310	176	326	175	154
284	188	340	197	209
304	185	337	189	210
364	180	340	123	118
339	149	327	188	129
317	135	331	171	124
318	172	335	163	175
320	182	331	161	175
341	181	317	166	180
301	189	358	174	156
291	183	332	170	164
299	188	292	164	158
292	162	314	171	174
293	164	324	178	179
302	208	344	206	182
291	175	316	194	181
316	181	295	213	190
296	200	320	182	183
304	194	305	197	182
295	139	298	195	158
354	146	310	183	170
323	162	308	137	167
338	150	314	132	154
319	123	303	169	171
327	166	303	194	188
343	171	369	189	174
319	162	327	191	119
332	161	348	195	170
333	155	339	182	183
268	164	343	180	206
312	156	318	184	164
270	159	295	164	157
346	174	296	157	138
315	195	294	128	174

data.Fig.S6f

Col-7	ckrc3	Col-0	nbc-1	ckrc3 nbc-1
9.1	5.9	9.1	6.4	6.1
8.5	4.9	8.5	6.0	4.5
8.2	5.4	8.2	6.6	5.4
8.6	5.4	8.6	6.3	5.3
10.5	8.8	8.2	7.5	6.1
7.3	10.4	10.1	6.2	5.5
9.1	9.1	7.9	7.1	6.5
9.2	4.9	10.5	5.7	5.5
11.1	7.6	10.4	6.7	5.6
9.5	9.1	7.4	4.5	5.4
9.5	6.3	9.8	6.2	4.8
10.1	7.7	8.9	4.4	4.6
8.5	8.4	7.4	5.8	5.5
7.2	4.4	8.4	4.1	4.8
6.6	5.5	8.1	3.7	3.3
7.0	8.1	5.4	5.3	6.5
8.6	5.2	9.5	6.3	4.3
9.9	5.4	10.0	6.1	5.1
7.4	4.6	7.8	7.0	3.1
7.1	8.5	8.0	4.9	5.3
8.5	6.0	5.9	5.0	4.4
10.6	7.9	8.8	6.9	4.8
8.5	6.3	12.1	6.0	5.0
8.6	5.6	9.9	7.1	5.7
7.1	7.2	8.8	6.0	5.5
9.5	7.0	9.8	7.0	5.7
9.9	6.0	7.0	7.2	6.9
9.2	5.2	7.5	5.1	4.1
8.5	6.0	5.8	5.9	6.6
12.4	5.2	8.5	7.0	4.7
10.8	5.8	7.6	7.5	6.1
12.4	4.9	7.9	7.2	6.7
11.3	3.7	9.8	6.4	4.3
10.7	6.4	6.8	7.2	6.8
4.5	6.0	6.2	6.5	8.0
7.7	7.0	6.2	6.4	5.8
10.7	6.5	6.2	8.6	5.4
8.4	4.9	6.1	6.2	5.1
8.3	4.0	8.2	5.2	2.5
8.9	5.6	9.7	6.3	4.6
9.1	6.3	7.7	5.0	3.3
11.1	3.8	9.5	5.7	3.4
8.0	4.5	5.9	8.9	4.3
8.0	4.6	5.9	6.3	3.8
8.2	3.2	9.8	5.2	4.4
11.5	3.1	8.9	6.0	5.2
9.3	3.9	8.9	4.4	4.9
8.1	5.5	12.6	5.2	4.9
6.8	5.3	7.6	4.1	5.1

6.1	4.1	8.4	6.9	6.8
7.0	3.1	8.9	6.8	4.7
8.7	3.1	8.9	9.3	4.7
11.1	4.2	10.8	7.2	5.7
9.7	4.9	9.5	6.7	4.7
10.1	4.5	9.0	4.4	5.5
7.8	4.9	7.2	6.2	6.4
7.8	4.5	7.3	7.5	4.1
7.4	4.1	7.7	7.6	5.0
8.2	5.1	9.4	9.3	5.4
10.0	4.1	7.8	4.7	5.5
9.6	3.9	11.5	4.5	6.2
10.5	2.4	9.5	7.5	6.2
6.5	3.4	11.4	5.9	6.5
7.3	4.5	8.0	6.3	5.2
8.8	5.8	10.6	6.6	7.1
8.9	5.1	6.7	7.4	7.1
7.6	4.0	7.4	6.9	6.6
7.6	3.8	9.8	5.8	5.2
7.3	3.3	9.2	8.0	6.8
6.5	3.1	8.6	6.7	6.5
6.8	4.7	8.4	7.3	5.4
6.6	4.8	7.5	7.2	4.3
7.4	4.3	9.2	8.7	4.6
6.8	5.6	7.8	6.0	5.8
10.4	5.6	7.4	5.2	5.2
8.8	4.7	7.8	4.9	6.1
7.2	6.3	7.9	6.3	5.1
8.9	7.0	7.2	5.7	5.1
6.9	4.7	9.9	7.2	4.7
8.0	4.9	10.6	7.4	5.8
7.5	5.3	10.1	6.7	4.7
7.2	5.5	8.1	5.1	4.9
8.3	3.7	9.7	8.9	4.3
8.9	4.0	8.5	6.1	5.1
8.2	7.1	8.6	7.3	5.2
7.9	6.0	7.9	5.8	5.0
8.2	5.8	8.1	4.5	6.6
8.1	5.0	7.3	5.4	5.4
7.9	5.2	9.5	6.6	4.8
				6.2

data.Fig.S10b

Col-7	1.09	1.01	0.91
ckrc3 nbc-1	1.22	0.95	1.06
35S::YUC8-eGFP/Col-7	26.28	25.92	27.35
35S::YUC8-eGFP/ckrc3 nbc-1	22.73	25.68	24.38

data.Fig.S10c

35S::YUC8-eGFP/Col-7	35S::YUC8-eGFP/ckrc3 nbc-1
440.264	64.358
597.365	147.929
645.066	229.873
379.226	119.002
795.154	157.771
594.238	143.284
779.366	204.813
449.298	50.336
499.87	181.235
505.321	168.132

data.Fig.S11c

CK1	0.0	0.0	0.0
CK2	34.8	35.7	37.1
YUC8	95.3	99.5	106.5
YUC8(E2A)	98.9	108.1	103.6

data.Fig.S13

Col	ckrc3	nbc-1	ckrc3 nbc-1
0.96311	2.259137	1.805106	2.415102
0.990186	2.306606	1.881762	2.222347
1.046645	2.338805	1.989056	2.176611
0.983879	2.405861	1.934092	1.734092
0.924377	2.372738	1.920306	1.871481
1.091682	2.389242	2.207732	1.832967
1.075221	2.416342	2.265566	1.996325
1.003621	2.555316	2.375323	1.967812
0.921158	2.316342	2.999985	2.082365
