# Supplementary material for: Significance of NatB-mediated N-terminal acetylation of auxin biosynthetic enzymes in maintaining auxin homeostasis in Arabidopsis thaliana
Source: Commun Biol. 2022 Dec 22;5:1410. doi: 10.1038/s42003-022-04313-9 (PMC9780221; doi:10.1038/s42003-022-04313-9)
Supplement: Supplementary file 5 — Reporting Summary [file 42003_2022_4313_MOESM5_ESM.pdf]

nature portfolio

Corresponding author(s): Guang-Qin Guo

Last updated by author(s): Jul 12, 2022

Reporting Summary

Nature Portfolio wishes to improve the reproducibility of the work that we publish. This form provides structure for consistency and transparency in reporting. For further information on Nature Portfolio policies, see our Editorial Policies and the Editorial Policy Checklist.

Statistics

For all statistical analyses, confirm that the following items are present in the figure legend, table legend, main text, or Methods section.

n/a	Confirmed	
☐	☒	The exact sample size (n) for each experimental group/condition, given as a discrete number and unit of measurement
☐	☒	A statement on whether measurements were taken from distinct samples or whether the same sample was measured repeatedly
☐	☒	The statistical test(s) used AND whether they are one- or two-sided<br>*Only common tests should be described solely by name; describe more complex techniques in the Methods section.*
☒	☐	A description of all covariates tested
☒	☐	A description of any assumptions or corrections, such as tests of normality and adjustment for multiple comparisons
☐	☒	A full description of the statistical parameters including central tendency (e.g. means) or other basic estimates (e.g. regression coefficient) AND variation (e.g. standard deviation) or associated estimates of uncertainty (e.g. confidence intervals)
☒	☐	For null hypothesis testing, the test statistic (e.g. F, t, r) with confidence intervals, effect sizes, degrees of freedom and P value noted<br>*Give P values as exact values whenever suitable.*
☒	☐	For Bayesian analysis, information on the choice of priors and Markov chain Monte Carlo settings
☒	☐	For hierarchical and complex designs, identification of the appropriate level for tests and full reporting of outcomes
☒	☐	Estimates of effect sizes (e.g. Cohen's d, Pearson's r), indicating how they were calculated

Our web collection on statistics for biologists contains articles on many of the points above.

Software and code

Policy information about availability of computer code

Data collection	No software was used.
Data analysis	No software was used.

For manuscripts utilizing custom algorithms or software that are central to the research but not yet described in published literature, software must be made available to editors and reviewers. We strongly encourage code deposition in a community repository (e.g. GitHub). See the Nature Portfolio guidelines for submitting code & software for further information.

Data

Policy information about availability of data

All manuscripts must include a data availability statement. This statement should provide the following information, where applicable:

- Accession codes, unique identifiers, or web links for publicly available datasets
- A description of any restrictions on data availability
- For clinical datasets or third party data, please ensure that the statement adheres to our policy

All data generated during this study are included in the supplementary information files.

Human research participants

Policy information about studies involving human research participants and Sex and Gender in Research.

Reporting on sex and gender	Not applicable
Population characteristics	Not applicable
Recruitment	Not applicable
Ethics oversight	Not applicable

Note that full information on the approval of the study protocol must also be provided in the manuscript.

Field-specific reporting

Please select the one below that is the best fit for your research. If you are not sure, read the appropriate sections before making your selection.

☒ Life sciences ☐ Behavioural & social sciences ☐ Ecological, evolutionary & environmental sciences

For a reference copy of the document with all sections, see nature.com/documents/nr-reporting-summary-flat.pdf

Life sciences study design

All studies must disclose on these points even when the disclosure is negative.

Sample size	no sample-size calculation was performed, the sample sizes used is described in figure legends in the manuscript, according to those commonly used in similar researches
Data exclusions	No data were excluded from the analyses.
Replication	Each experiment was repeated at least 3 times, and the results were also confirmed by different test, such as the low auxin phenotypes and the Dr5-Gus/GFP observation.
Randomization	The samples were used in this study are randomly selected.
Blinding	The investigators were blinded to group allocation during data collection and/or analysis.

Reporting for specific materials, systems and methods

We require information from authors about some types of materials, experimental systems and methods used in many studies. Here, indicate whether each material, system or method listed is relevant to your study. If you are not sure if a list item applies to your research, read the appropriate section before selecting a response.

Materials & experimental systems

n/a	Involved in the study	
☐	☒	Antibodies
☒	☐	Eukaryotic cell lines
☒	☐	Palaeontology and archaeology
☒	☐	Animals and other organisms
☒	☐	Clinical data
☒	☐	Dual use research of concern

Methods

n/a	Involved in the study	
☒	☐	ChIP-seq
☒	☐	Flow cytometry
☒	☐	MRI-based neuroimaging

Antibodies

Antibodies used	anti-GFP monoclonal antibody (1:5,000; Abmart, Catalog no. M20004); anti-Plant Actin monoclonal antibody (1:5000; Abbkine, Catalog no. ABL1050); Anti-His polyclonal antibody (1:5000; Solarbio, Catalog no. k007439p); anti-Myc monoclonal antibody (1:5000; Novogene, Catalog no. NHT0044); anti-HA polyclonal antibody (1:5000; Proteintech, Catalog no. 51064-2-AP).
Validation	The anti-GFP monoclonal antibody is produced by immunizing mouse with full length recombinant GFP, and was used to detect GFP and GFP fusion proteins. The anti-Plant Actin monoclonal antibody is produced by immunizing mouse with Recombinant Actin, and

was used to detect Actin in Arabidopsis, Brassica napus, Corn. The anti-His polyclonal antibody is produced by immunizing rabbits with synthetic peptide (6×His), and was used to detect fusion proteins containing the HIS Tag. The anti-Myc monoclonal antibody is produced by immunizing rabbits with synthetic peptide (410-419 of human c-Myc (EQKLISEEDL)), and was used to detect fusion proteins containing the myc Tag. The anti-HA polyclonal antibody is produced by immunizing rabbits with synthetic peptide ((YPYDVPDYA)), and was used to detect fusion proteins containing the HA Tag.
